# Promising Metabolite Profiles in the Plasma and CSF of Early Clinical Parkinson's Disease

**DOI:** 10.3389/fnagi.2018.00051

**Published:** 2018-03-05

**Authors:** Daniel Stoessel, Claudia Schulte, Marcia C. Teixeira dos Santos, Dieter Scheller, Irene Rebollo-Mesa, Christian Deuschle, Dirk Walther, Nicolas Schauer, Daniela Berg, Andre Nogueira da Costa, Walter Maetzler

**Affiliations:** ^1^Metabolomic Discoveries GmbH, Potsdam, Germany; ^2^Department of Biochemistry and Biology, Universität Potsdam, Potsdam, Germany; ^3^Max Planck Institute für Molekulare Pflanzenphysiologie, Potsdam, Germany; ^4^Department of Neurodegeneration, German Center for Neurodegenerative Diseases, Hertie Institute for Clinical Brain Research, University of Tuebingen, Tuebingen, Germany; ^5^Experimental Medicine and Diagnostics, Global Exploratory Development, UCB Biopharma SPRL, Brussels, Belgium; ^6^Consultancy Neuropharm, Neuss, Germany; ^7^Exploratory Statistics, Global Exploratory Development, UCB Pharma SA, Slough, United Kingdom; ^8^Department of Neurology, Christian-Albrechts-University Kiel, Kiel, Germany

**Keywords:** biomarker, untargeted metabolomics, neurodegeneration, plasma, CSF, machine learning

## Abstract

Parkinson's disease (PD) shows high heterogeneity with regard to the underlying molecular pathogenesis involving multiple pathways and mechanisms. Diagnosis is still challenging and rests entirely on clinical features. Thus, there is an urgent need for robust diagnostic biofluid markers. Untargeted metabolomics allows establishing low-molecular compound biomarkers in a wide range of complex diseases by the measurement of various molecular classes in biofluids such as blood plasma, serum, and cerebrospinal fluid (CSF). Here, we applied untargeted high-resolution mass spectrometry to determine plasma and CSF metabolite profiles. We semiquantitatively determined small-molecule levels (≤1.5 kDa) in the plasma and CSF from early PD patients (disease duration 0–4 years; *n* = 80 and 40, respectively), and sex- and age-matched controls (*n* = 76 and 38, respectively). We performed statistical analyses utilizing partial least square and random forest analysis with a 70/30 training and testing split approach, leading to the identification of 20 promising plasma and 14 CSF metabolites. These metabolites differentiated the test set with an AUC of 0.8 (plasma) and 0.9 (CSF). Characteristics of the metabolites indicate perturbations in the glycerophospholipid, sphingolipid, and amino acid metabolism in PD, which underscores the high power of metabolomic approaches. Further studies will enable to develop a potential metabolite-based biomarker panel specific for PD.

## Introduction

PD is the second most common neurodegenerative disorder after Alzheimer's disease (AD) and the most common form of neurodegenerative movement disorders with about 5 million affected worldwide (Dorsey et al., 2007). The disease is primarily caused by a progressive degeneration of dopaminergic neurons, e.g., in the substantia nigra pars compacta (Poewe et al., 2017), leading to various motor symptoms such as bradykinesia, rigidity, rest tremor, and postural instability (Lang and Lozano, 1998; Diaz and Waters, 2009; Xia and Mao, 2012). Non-motor symptoms, such as loss of sense of smell, sleep disturbances, constipation, cognitive deficits, and depression, are also regularly observed. Clinical diagnosis is based on the detection of a combination of cardinal motor symptoms including bradykinesia and rigidity (Postuma et al., 2015).

As current diagnostic approaches still lead to a high proportion of misdiagnoses, in particular, at the early disease stages (Poewe et al., 2017), biochemical markers that inform on disease PD pathogenesis are needed. Preferably, these biomarkers should be accessible in non- or low-invasive samples such as blood, saliva, cerebrospinal fluid (CSF), or urine (Wang et al., 2013), and reflect the underlying molecular mechanisms of the disease. PD pathogenesis involves multiple pathways and mechanisms, such as alpha-synuclein- (Spillantini et al., 1998), tau (Lei et al., 2010), and amyloid beta (Gomperts et al., 2008) misfolding, mitochondrial dysfunction (Schapira, 2008), oxidative stress (Blesa et al., 2015), calcium dyshomeostasis (Rivero-Ríos et al., 2014), axonal transport deficits (Lamberts et al., 2015), and neuroinflammation (Wang et al., 2015). Using the above-mentioned biofluids, researchers have begun to adapt non-hypothesis-driven system biology “omics” approaches, such as metabolomics, to investigate novel biomarkers that provide fingerprints linked to PD diagnosis and molecular pathogenesis. Previous studies point to involvement of various pathways, such as glutathione- (Bogdanov et al., 2008; Lewitt et al., 2013; Trupp et al., 2014), lipid- (Garcia-Sanz et al., 2017), purine- (Bogdanov et al., 2008; Johansen et al., 2009; Luan et al., 2015a,b; LeWitt et al., 2017), energy- (Ahmed et al., 2009; Trupp et al., 2014; Ohman and Forsgren, 2015), polyamine- (Roede et al., 2013), tryptophane/kynurenine- (Lewitt et al., 2013; Trupp et al., 2014; Luan et al., 2015a,b; Hatano et al., 2016; Havelund et al., 2017), fatty acid- and beta oxidation- (Trupp et al., 2014; Wuolikainen et al., 2016; Burte et al., 2017; LeWitt et al., 2017; Saiki et al., 2017), phenylalanine- (Hatano et al., 2016), and histidine (Burte et al., 2017) metabolisms. Metabolomics based studies on potential markers for early PD diagnosis could identify increased levels of fructose, mannose, und threonic acid and decreased levels of dehydroascorbic acid in PD patients (Trezzi et al., 2017). A detailed list of the current state of metabolomics research in PD can be found in Supplementary Table 3.

Metabolomics is a rapidly evolving high-throughput technology that allows measuring the entire complement of metabolites, typically in a mass range of 50–1,700 Da, in complex samples such as biological fluids or tissues (Patti et al., 2012). High-resolution mass spectrometry (HRMS) in combination with liquid chromatography (LC) enables simultaneous semi-quantitative measurements of various molecular species such as amino acids, nucleotides, carbohydrates, peptides, and various lipids. Therefore, metabolomics is a suitable technology to obtain a comprehensive overview of the functional state of the organism (Zhou et al., 2012; Want et al., 2013; Contrepois et al., 2015; Ivanisevic and Thomas, 2018) by reflecting the complex interaction of genes, proteins, and the surroundings. Metabolome measurement informs about the complex network of metabolic interactions that collectively define a phenotypic state (Ravasz et al., 2002; Michell et al., 2008).

By analyzing blood plasma and CSF using HRMS metabolomics (Hatano et al., 2016; Sanyal et al., 2016), our study set out to discover metabolic profiles that allow the differentiation of PD from control state and to gain insight into the molecular pathogenesis of the disease. Blood plasma and CSF were used to investigate whether findings can be translated from one matrix to the other (CSF to plasma) and therefore increasing the possibility of moving to a prodromal investigation of PD. By utilizing machine learning algorithms, such as partial least square (PLS) (Land et al., 2011) and tree-based ensemble random forest (RF) approaches (Breiman, 2001), we identified a panel of 20 metabolites in plasma and 14 metabolites in CSF that enabled us to distinguish PD from controls with high accuracy.

## Materials and methods

### Patient recruitment and diagnosis

PD diagnosis was based on the UK Brain Bank Society's criteria for Parkinson's disease (Hughes et al., 1992). All controls were thoroughly assessed as having no neurological disease. To represent a homogeneous as possible cohort with very early disease state (biomaterial withdrawal 0–4 years after disease diagnosis), only patients with akinetic-rigid and equivalent subtype were included. All participants underwent a clinical assessment and provided plasma and a subcohort provided CSF samples in the course of clinical routine assessment and prospective studies. These samples were stored in the local biobank of the Neurological Department of the University Medical Center Tübingen (see below). All participants provided written informed consent and the study was approved by the local ethical board. The main demographic and clinical features of the cohorts are summarized in Table 1. Note that the cohorts were well balanced with regard to age and gender frequency. All PD patients included in this study were tested negative for the most frequent known pathogenic mutations involved in PD (*LRRK2* G2019S, *GBA* L444P, and N370S) and none of the patients were dyskinetic (early disease stage).

**Table 1:** Demographic and clinical features of patients with Parkinson's disease and controls.

	**Plasma**		**CSF**	
	**PD**	**Controls**	**PD**	**Controls**
Males/Females [N]	54/26	48/28	24/16	25/12
Age [y], median (IQR)	66 (12)	65 (17)	67 (14)	66 (14)
Disease duration [y], median	3 (2)	/	3 (1)	/
LEDD, median (IQR)	208 (317)	/	160 (353)	/
HY, median (range)	2 (1–4)	/	2 (1–4)	/
MMSE, median (IQR)	29 (2)	30 (1)	29 (3)	30 (1)
MoCA, median (IQR)	27 (4)	28 (3)	27 (4)	28 (3)
UPDRS (3), median (IQR)	21 (13)	0 (2)	23 (15)	0 (1)
BDI, median (IQR)	8 (9)	2 (4)	7 (5)	2 (4)

*PD, Parkinson's disease; IQR, interquartile range (Q3–Q1); LEDD, L-Dopa equivalent daily dose; HY, Hoehn and Yahr scale; MMSE, Mini Mental State Examination; MoCA, Montreal Cognitive Assessment; UPDRS, Unified Parkinson's Disease Rating Scale; BDI, Beck Depression Inventory*.

### Sample collection, storage, and preparation

For this study, we obtained plasma samples from 80 PD patients and 76 sex- and gender-matched controls, and CSF samples from 40 PD patients and 37 sex- and gender-matched controls. The biobank of the Neurological Department of the University Medical Center Tübingen fulfills the highest international standards regarding sample collection, processing and storage (Maetzler et al., 2011; Reijs et al., 2015). Briefly, samples were collected after overnight fasting between 8:00 and 11:00 am. Peripheral blood was collected in S-Monovette® 9 ml, K3 EDTA, 92 × 16 mm test tubes (Sarstedt, 02.1066.001) and centrifuged for 10 min at 2,000 × g at 4°C. CSF was collected and centrifuged for 10 min at 2,000 × g at room temperature. The supernatants were aliquoted and stored at −80°C until further analysis. The complete processing from sample withdrawal to storage did not exceed 90 min. All samples were thawed for the first time at the site of investigation and directly processed on ice within 2 h. Metabolites were extracted using 90% MeOH and10% water spiked with internal standards with constant shaking for 15 min at 37°C (1000 rpm) followed by centrifugation to remove the precipitate and the supernatant transferred into LC/MS vials.

### LC/MS and MS/MS analysis

Modified hydrophilic interaction chromatography (pHILIC) was employed in combination with HRMS. Samples were pseudonymised twice and third party concealment of the origin of respective specimens (controls or PD) was achieved by using uniquely coded vials. Samples were randomized on an Agilent 1290 UHPLC system (Agilent, Santa Clara, USA) with a ZIC-pHILIC column (10 cm × 2.1 mm, 3 μm, Sequant, Merck) coupled to a high-resolution 6540 QTOF/MS detector (Agilent, Santa Clara, USA) operated in positive ESI mode in a detection range of 50–1700 *m/z* at 2 GHz in extended dynamic range. The LC solvent consisted of (A) 95% 20 mM ammonium carbonate with 5% acentonitrile pH 9 and (B) 95% acetonitrile with 5% 20 mM ammonium carbonate with a multi-step gradient with 5% B from 0–1 min, then to 35% B at 8.5 min, to 95% B at 9.5 min kept constant until 12 min, to 5% B at 12.01 min and washing until 15 min with 5% B. The flow rate was kept constant at 300 μl/min. The total run time was 15 min, 1 μl of plasma sample, and 2 μl of CSF sample were injected and the column heated to 30°C. The DualAJS ESI source was set to the following parameters: gas temperature 200°C, drying gas 8 l/min, nebulizer 35 psi, sheath gas temp: 350°C, sheath gas flow 11 l/min, VCAp 3.5 kV and nozzle voltage of 0 V. Online calibration of the instrument was performed throughout the data acquisition using the Agilent ESI-TOF Reference Mass Solution Kit. We acquired MS/MS spectra in positive and negative ionization modes. Analyte stability, signal reproducibly, and chromatographic peaks were monitored by biological quality controls, which were analyzed periodically throughout the sample batches.

### Metabolomics data analysis

Raw data were converted to mzXML and chromatogram peaks extracted with XCMS (Smith et al., 2006), which were optimized by using the IPO R-package (Libiseller et al., 2015). Mzmatch.R was used for peak filtering based on minimum detectable intensity (2000), peak shape filtering (codadw > 0.9) and for the annotation of related peaks (Scheltema et al., 2011). Missing peaks ≤10% were computed using Bayesian PCA based estimation measures (Oba et al., 2003). Additional filtering was performed by excluding peaks with lower median peak intensities per group in biological samples compared to blanks (extraction solvent only). The remaining data was normalized based on multiple internal standards applying NOMIS (Sysi-Aho et al., 2007) and CCMN (Redestig et al., 2009) normalization followed by mean total ion chromatogram (TIC) normalization. IDEOM software was used (http://mzmatch.sourceforge.net/ideom.php) (Creek et al., 2012) to eliminate noise and artifacts and for putative peak annotation by exact mass within ± 10 ppm against the Metabolomic Discoveries in-house metabolite library in positive ESI mode. Retention time prediction was applied (Creek et al., 2011) to aid metabolite annotation and identities confirmed by available authentic standards (validation level 1). MS/MS spectra were matched against online databases such as Metlin and MassBank (validation level 2) or against *in silico* fragmentation spectra (validation level 3) retrieved from Metfrag (Ruttkies et al., 2016), CFM-ID (Allen et al., 2014) and/or CSI:FingerID (Dührkop et al., 2015) with precursor mass accuracy of 20 ppm and fragment accuracy of 0.01 Da. Semi-quantitative metabolite intensities were calculated using the raw peak height.

### Statistical analysis

From the initial sample set of 80 plasma and 40 PD CSFs, and 76 plasma and 37 control CSF samples, outlier samples were identified using ROBPCA (Hubert et al., 2005) by defining the sample distances within the orthogonal to the projection plane. As a consequence, in plasma, 10 outliers (6.4% of all plasma samples; seven controls, five males, two females; three male PD patients) were removed from the initial dataset (Supplementary Figure 1). In CSF, one female control and one male PD patient were removed (2.6% of all CSF samples). Of note, study participants from whom samples have been drawn, did not show any other specific diseases and were not differently treated than the remaining cohorts. A potential L-Dopa medication bias was addressed by linear regression of L-Dopa dose/response relationships and measured metabolite levels. Age correction was performed using linear age/metabolite level normalization including PD and controls. In detail, linear regression based on the dependency of age and metabolite level was applied. Metabolites showing a significant correlation, e.g., slope (*p* ≤ 0.05), were normalized to slope = 0. Potential gender bias was corrected by normalizing to equal mean metabolite levels in males and females including all PD and control samples. Interactions between age, gender, metabolite level and L-Dopa dosage were found not to be significant (ANOVA, *p* > 0.05). PLSs, as used in this study, reduces the set of metabolites to a smaller set of uncorrelated components with maximal co-variance to the target variable(s) and performs least squares regression on these (minimization of the sum of squared errors). This supervised approach combines features of principal component analysis and multiple linear regressions to determine the most discriminatory metabolites between different classes, here PD and control subjects. The second approach used in this study, Random Forest (RF), is a tree-based approach, which trains multiple decision trees on bootstrap sample derived from the original dataset with subsequent performance evaluation on the left out samples. The predictions of all trees are then combined via majority vote. In RF, the frequency at which individual variables (metabolites) are used in the individual trees can be interpreted as their relevance in the decision process. Ten times 10-fold cross validated PLS and RF models were built using caret R package (Kuhn, 2008). First, the full data set was randomly split into 70% of samples for model training and 30% of samples for model testing for the plasma and CSF cohort, respectively.

For this sample selection, the RF model was tuned (mtry tuning, with lowest out of bag error) by building 1,000 trees using the random forest R package (Liaw and Wiener, 2002). Initial PLS models were built using a maximum number of 20 components to determine the maximum number of components needed to achieve the largest Area Under the ROC Curve (AUC-ROC). To compare the performance (based on model training AUC-ROC) of the cross-validated PLS and RF models, the 70/30 training and testing split was repeated 200 times, allowing also the assessment of model robustness. For the final feature selection, ranked scaled variables in the projection (VIP) scores of the top 100 metabolites were plotted and a selection threshold was established based on the point in the resulting curve where the slope flattens. These metabolites were used to train the final cross-validated PLS and RF models, which were tested with the remaining (single split) 30% of blinded samples in plasma and CSF, respectively. Extracted informative metabolites from the best-performing model (either PLS or RF) were considered for further analysis. To test, whether all of the selected metabolites were needed to achieve the highest AUC and accuracy, PLS Monte-Carlo cross validation using balanced subsampling was performed on the selected metabolites in plasma and CSF. In each cross-validation, two third of the samples were used to evaluate the metabolite importance, the top metabolites were then used to build a classification model, which is validated on one third of the samples that were left out. These calculations were carried out utilizing MetaboAnalyst (Xia et al., 2015).

**Figure 1:**
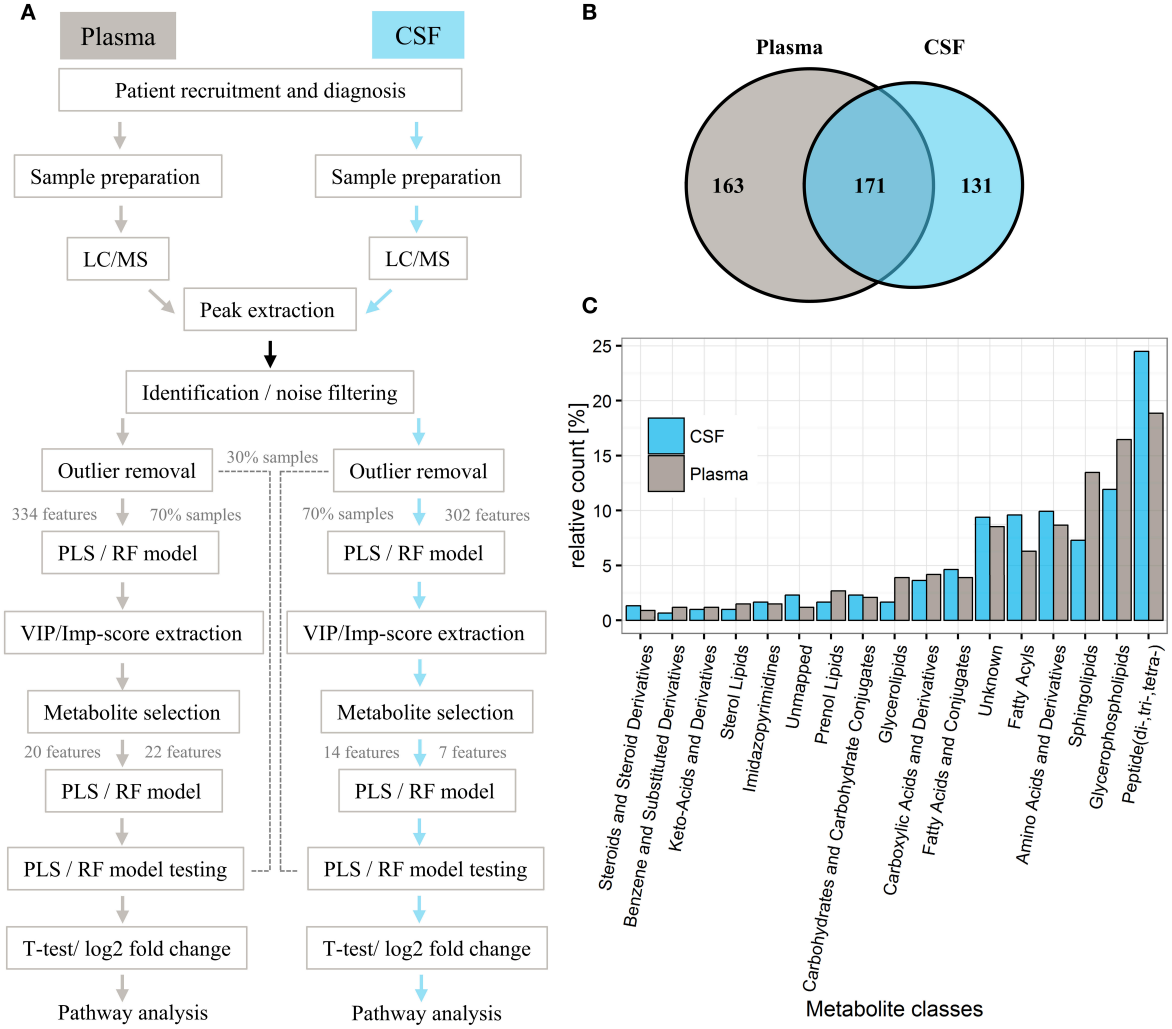
General workflow for investigating metabolic profiles of PD patients and controls and respective distribution of metabolites in plasma and CSF. **(A)** General workflow of data analysis for samples from controls and PD patients. **(B)** Venn diagram of metabolites detected in respective plasma and CSF sets. **(C)** Bar chart illustrating the relative count of putatively identified metabolites from each metabolite class, classified according to KEGG, Lipidmaps, and HMDB. In total, 334 metabolites were analyzed in plasma and 302 metabolites analyzed in CSF (for a full list of extracted metabolites, see Supplementary Table 1). The identified metabolite classes (relative count > 5%) included (di-, tri-, tetra-) peptides (18% plasma, 25% CSF), glycerophospholipids (plasma 17%, CSF 12%), sphingolipids (plasma 14%, CSF 7%), amino acids and derivatives (plasma 9%, CSF 10%), fatty acyls (plasma 6%, CSF 10%), and unknowns (8% plasma, 9% CSF; no match to our metabolite database).

Normal distribution of the selected metabolites was tested utilizing the Shapiro-Wilks test (α = 0.05) (Royston, 1995) and metabolites compared between cases (PD vs. controls) using a two-sided Welch's *t*-test or Wilcoxon-Cox test. *P*-values were corrected for multiple testing using Benjamini and Hochberg (BH) false discovery rate (FDR) adjustment (Benjamini and Hochberg, 1995). Univariate AUC measures and 95% confidence intervals (CI) were calculated for the selected metabolites using 500 bootstrappings via MetaboAnalyst (Xia et al., 2015). Significant levels in MetaboAnalyst pathway analysis were based on hypergeometric tests and the pathway impact values determined by relative-betweeness centrality (Xia et al., 2015). For a detailed illustration of the workflow of this study see Figure 1A.

## Results

### Plasma and CSF metabolites distinguish PD patients from controls

Overall, 2,130 and 1,798 peaks were present in the plasma and CSF sample sets, respectively. Successive noise filtering and putative peak annotation resulted in the nomination of 334 metabolites in plasma and 302 in CSF, with most metabolites (171) detected in both compartments (Figure 1B, Supplementary Table 1) from various metabolite classes (Figure 1C). The within experiment technical and analytical variations were monitored by periodic analysis of biological quality controls. These showed a median relative standard deviation (RSD) of < 15%, which is well within acceptable limits for metabolomics (Kirwan et al., 2014).

### PLS model separates controls from PD based on metabolites in both plasma and CSF with high sensitivity

**Figure 2:**
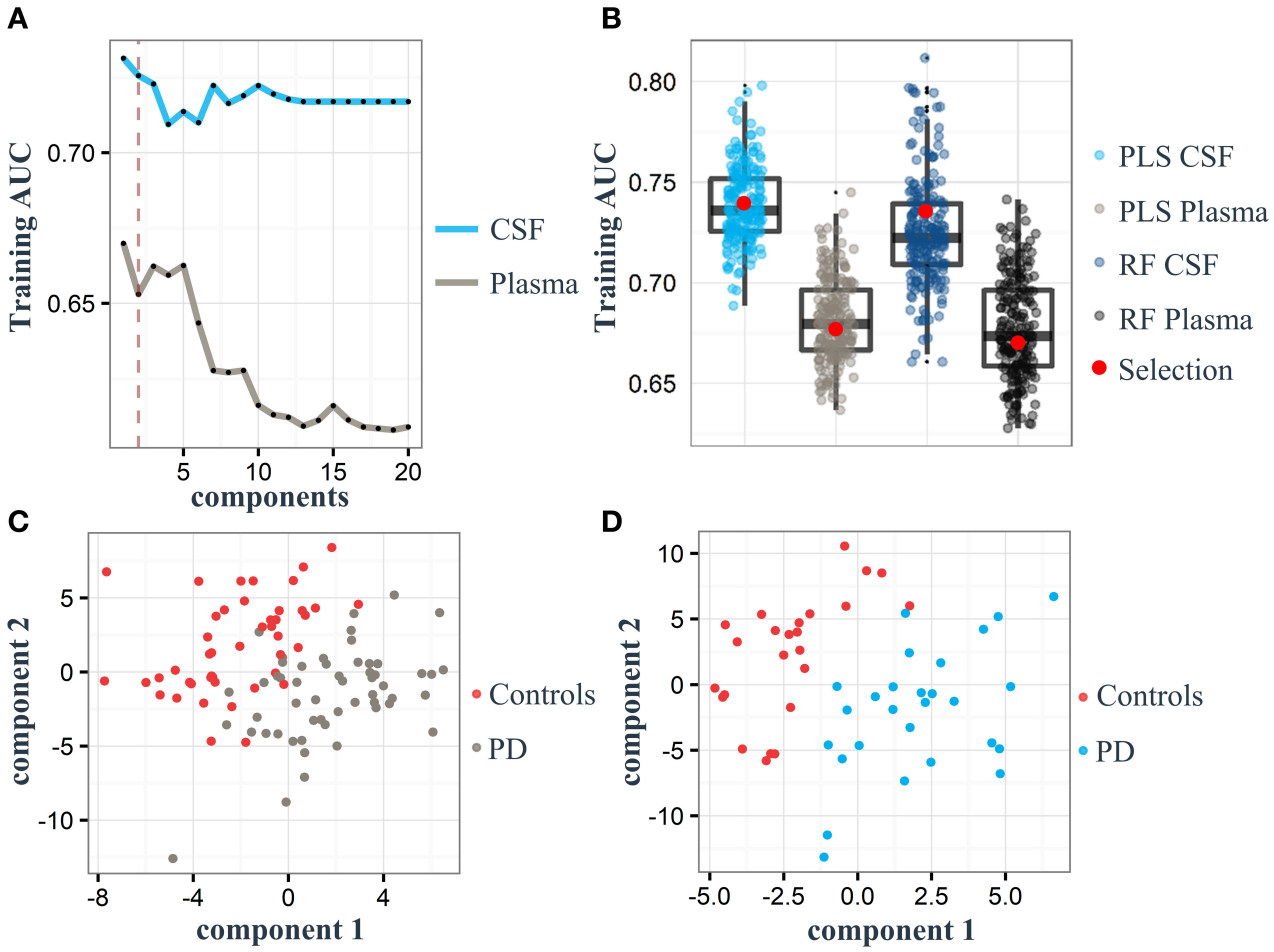
Partial least square (PLS) component plots and the associated separation of Parkinson's disease (PD) and control status based on plasma and cerebrospinal fluid (CSF) samples. **(A)** Components and their corresponding area under the curve (AUC; gray: plasma, light blue: CSF). Dashed red line represents the maximum of two components used for model training in both cohorts. **(B)** Boxplots represent the training AUC of 200 randomly split samples (70% of the entire dataset) using PLS and random forest (RF) analyses. Red dot: Performance of samples selected for model training. **(C)** PLS score plot of all 334 metabolites identified in the plasma training set. **(D)** PLS score plot of all 302 metabolites identified in the CSF training cohort.

For both the plasma and CSF training sets, only two components were necessary to build PLS models with an AUC of 0.67 and 0.73, respectively (Figure 2A). Based on our random selection of training and test sets for plasma and CSF, results show that the initial random selection is well within the quantile range of other random selections (Figure 2B). This allowed us to reproducibly discriminate controls from PD samples in both the plasma and CSF training cohorts using a two-component PLS model, indicating strong metabolite signals in the plasma and CSF cohorts as also indicated by a clear separation of controls from PD in the PLS score plots obtained for the plasma and CSF samples, respectively (Figures 2A,C,D). Overall, the best PLS model generated for plasma samples yielded a mean AUC of 0.68 (95% CI = 0.67–0.68), mean sensitivity of 0.50 (95% CI = 0.49–0.51) and mean specificity of 0.66 (95% CI = 0.66–0.67). The PLS model obtained for CSF samples showed a mean AUC performance with a value of 0.74 (95% CI = 0.73–0.74), mean sensitivity of 0.44 (95% CI = 0.42–0.45) and mean specificity of 0.54 (95% CI = 0.53–0.55). In both plasma and CSF sets, PLS models performed similarly as RF [mean AUC for plasma = 0.68 (95% CI = 0.67–0.68), mean AUC for CSF = 0.73 (95% CI = 0.72–0.73)].

### Specific plasma- and CSF metabolites distinguish effectively PD patients from controls

**Figure 3:**
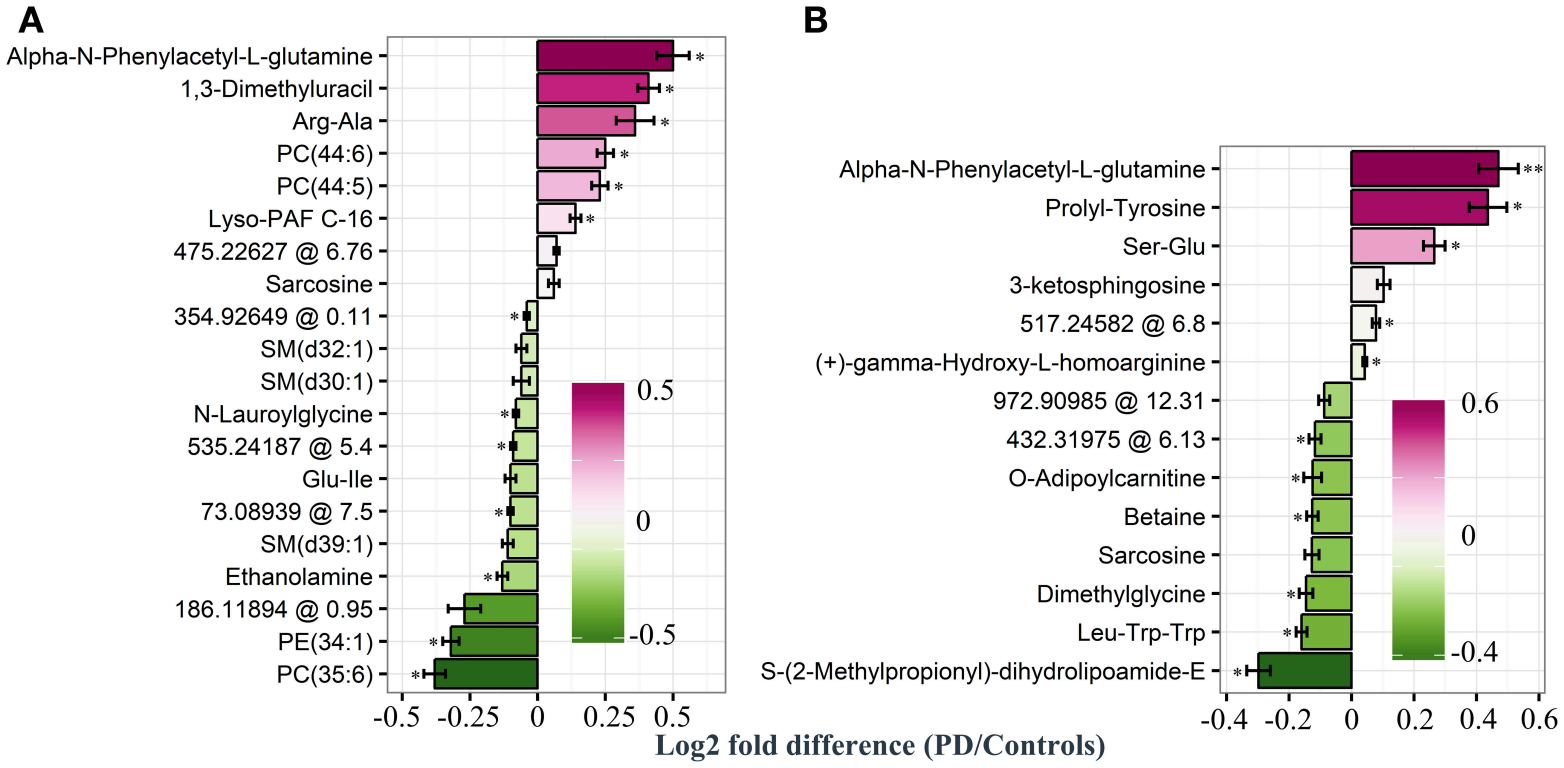
Log2 fold differences for top-ranked plasma and cerebrospinal (CSF) metabolites to differentiate early Parkinson's disease (PD) from controls, determined by the partial least square (PLS) model. Log2 fold differences (PD vs. controls) between values of metabolites retrieved from the plasma **(A)** and the CSF PLS model **(B)**. Red columns indicate higher values in PD. ^*^P < 0.05 and ^**^ < 0.01 according to univariate Welch's t- or Wilcoxon test (*p* < 0.05) after false discovery rate (FDR) correction by Benjamini and Hochberg (BH). Error bars indicate the standard error of the metabolite measure intensities.

Based on our PLS and RF models used for the differentiation of respective plasma and CSF samples, we extracted metabolites which contributed significantly to the differentiation between PD and controls. We selected 20 metabolites using the plasma PLS model (Figure 3A, Supplementary Figures 2, 3), of which 10 also ranked high in the RF model (22 important metabolites in the RF model overall, Table 2 and Supplementary Figure 4). From the CSF data, we selected 14 metabolites based on the PLS model (Figure 3B, Supplementary Figures 5, 6). Five of these metabolites were also considered important in the RF model (seven important metabolites in the RF model overall, Table 3 and Supplementary Figure 7). Interestingly, the CSF feature annotated as leu-trp-trp (C_28_H_33_N_5_O_4_) consistently ranked first in the PLS and second in the RF model. The metabolites annotated as sarcosine (C_3_H_7_NO_2_) and alpha-N-phenylacetyl-L-glutamine (C_13_H_16_N_2_O_4_) were found to be different between PD patients and controls in both compartments, plasma and CSF. Of note, sarcosine (C_3_H_7_NO_2_) showed a different trend in plasma (lower in PD compared to controls) than in CSF (higher in PD compared to controls).

**Table 2:** Detailed information on significantly changed metabolites between controls and PD patients in plasma retrieved from the PLS model.

**Proposed Metabolite**					**p**						
	**Mean**	**Mean**	**RSD**	**Log2 FD**	**Value**	**Rank**	**Rank**	**RF**			**Validation**
	**Co**	**PD**	**[%]**	**PD/Co**	**FDR**	**PLS**	**RF**	**Selected**	**AUC**	**95% CI**	**level**
73.08939 @ 7.5	3.56E+03	3.33E+03	18.22	−0.10	0.045	12	56	No	0.62	0.53–0.71	/
Ethanolamine	1.26E+04	1.15E+04	20.22	−0.13	0.019	10	35	No	0.63	0.54–0.73	1
475.22627 @ 6.76	3.27E+03	3.44E+03	15.23	0.07	0.079	13	66	No	0.58	0.48–0.68	/
N-Lauroylglycine	2.59E+03	2.45E+03	9.55	−0.08	0.012	3	47	No	0.66	0.56–0.75	3
Alpha-N-Phenylacetyl-L-glutamine	2.11E+04	2.98E+04	85.53	0.50	0.014	14	33	No	0.65	0.56–0.73	3
PC(35:6)	1.98E+04	1.52E+04	57.06	−0.38	0.012	20	118	No	0.64	0.55–0.74	2
Sarcosine	8.01E+05	8.36E+05	17.06	0.06	0.174	16	121	No	0.55	0.45–0.64	1
SM(d30:1)	6.69E+03	6.40E+03	28.79	−0.0624	0.397	17	40	No	0.56	0.46–0.65	2
SM(d32:1)	1.94E+05	1.86E+05	22.44	−0.0574	0.29	18	27	No	0.56	0.47–0.65	2
SM(d39:1)	2.25E+04	2.09E+04	29.20	−0.1101	0.128	11	25	No	0.59	0.48–0.68	2
Glu-Ile	5.45E+03	5.07E+03	20.73	−0.1038	0.059	7	1	Yes	0.6	0.51–0.69	2
535.24187 @ 5.4	4.13E+03	3.89E+03	12.67	−0.0853	0.014	5	8	Yes	0.64	0.55–0.74	/
186.11894 @ 0.95	2.38E+04	1.98E+04	66.10	−0.2669	0.104	15	18	Yes	0.59	0.49–0.69	/
1,3-Dimethyluracil	4.08E+03	5.42E+03	62.62	0.40917	0.014	6	9	Yes	0.63	0.52–0.72	3
PC(44:5)	1.15E+04	1.35E+04	36.99	0.23385	0.014	8	11	Yes	0.64	0.54–0.73	3
PC(44:6)	7.41E+03	8.84E+03	33.55	0.25355	0.012	1	6	Yes	0.66	0.58–0.74	2
PE(34:1)	3.52E+03	2.82E+03	41.11	−0.3214	0.014	2	10	Yes	0.64	0.54–0.72	3
Arg-Ala	1.00E+04	1.29E+04	69.02	0.35912	0.045	4	2	Yes	0.62	0.54–0.71	2
Lyso-PAF C-16	2.53E+04	2.79E+04	19.11	0.14038	0.012	9	3	Yes	0.65	0.57–0.73	2
354.92649 @ 0.11	4.37E+04	4.24E+04	6.58	−0.0435	0.04	19	4	Yes	0.63	0.53–0.72	/

*Significant ions detected between controls and Parkinson's disease (PD) patients. Proposed metabolite: proposed metabolite for each ion. If no formula could be calculated the proton corrected masses are reported with their corresponding retention time separated by a “@”. Log2 Fold difference (FD): relative abundance of mean of corresponding ion in PD compared to the mean of controls patients (Co). P value: value for unpaired Welch's t-test or Wilcoxon test FDR adjusted. AUC, Area under the curve; CI, 95% confidence interval; RSD, relative standard deviation*.

**Table 3:** Detailed information on significantly changed metabolites between controls and PD patients in CSF retrieved from the PLS model.

**Proposed Metabolite**					**P**						
	**Mean**	**Mean**	**RSD**	**Log2 FD**	**Value**	**Rank**	**Rank**	**RF**			**Validation**
	**Co**	**PD**	**[%]**	**PD/Co**	**FDR**	**PLS**	**RF**	**Selected**	**AUC**	**95% CI**	**level**
Prolyl-Tyrosine	6.99E+03	9.46E+03	53.98	0.44	0.045	9	30	No	0.66	0.55–0.79	3
Sarcosine	2.14E+04	1.96E+04	19.72	−0.13	0.111	12	51	No	0.62	0.49–0.75	1
Ser-Glu	7.46E+03	8.97E+03	39.14	0.26	0.062	10	26	Yes	0.62	0.49–0.74	2
432.31975 @ 6.13	6.45E+03	5.95E+03	16.59	−0.12	0.059	4	18	No	0.65	0.52–0.77	/
Leu-Trp-Trp	6.16E+03	5.51E+03	14.66	−0.16	0.026	1	2	Yes	0.7	0.59–0.82	2
Alpha-N-Phenylacetyl-L-glutamine	2.56E+04	3.55E+04	56.61	0.47	0.010	3	3	Yes	0.74	0.61–0.85	2
Betaine	4.95E+05	4.54E+05	15.93	−0.13	0.045	8	7	Yes	0.67	0.55–0.79	1
517.24582 @ 6.8	7.03E+03	7.42E+03	10.32	0.08	0.062	5	17	No	0.65	0.52–0.77	/
S-(2-Methylpropionyl)-dihydrolipoamide-E	7.35E+03	5.98E+03	37.10	−0.30	0.045	7	4	Yes	0.68	0.54–0.80	3
3-ketosphingosine	6.40E+03	6.87E+03	17.37	0.10	0.111	11	12	No	0.62	0.48–0.76	2
972.90985 @ 12.31	5.21E+03	4.91E+03	12.33	−0.09	0.075	2	5	No	0.63	0.50–0.76	/
(+)-gamma-Hydroxy-L-homoarginine	6.30E+04	6.49E+04	5.43	0.04	0.045	6	11	No	0.67	0.54–0.80	2
O-Adipoylcarnitine	5.96E+03	5.46E+03	25.71	−0.12	0.059	13	9	No	0.65	0.53–0.76	3
Dimethylglycine	8.06E+04	7.29E+04	17.91	−0.15	0.034	14	16	No	0.67	0.54–0.78	1

*Significant ions detected between controls and Parkinson's disease (PD) patients. Proposed metabolite: proposed metabolite for each ion. If no formula could be calculated the proton corrected masses are reported with their corresponding retention time separated by a “@”. Log2 Fold difference (FD): relative abundance of mean of corresponding ion in PD compared to the mean of controls patients (Co). P value: value for unpaired Welch's t-test or Wilcoxon test FDR adjusted. AUC, Area under the curve; CI, 95% confidence interval; RSD, relative standard deviation*.

**Figure 4:**
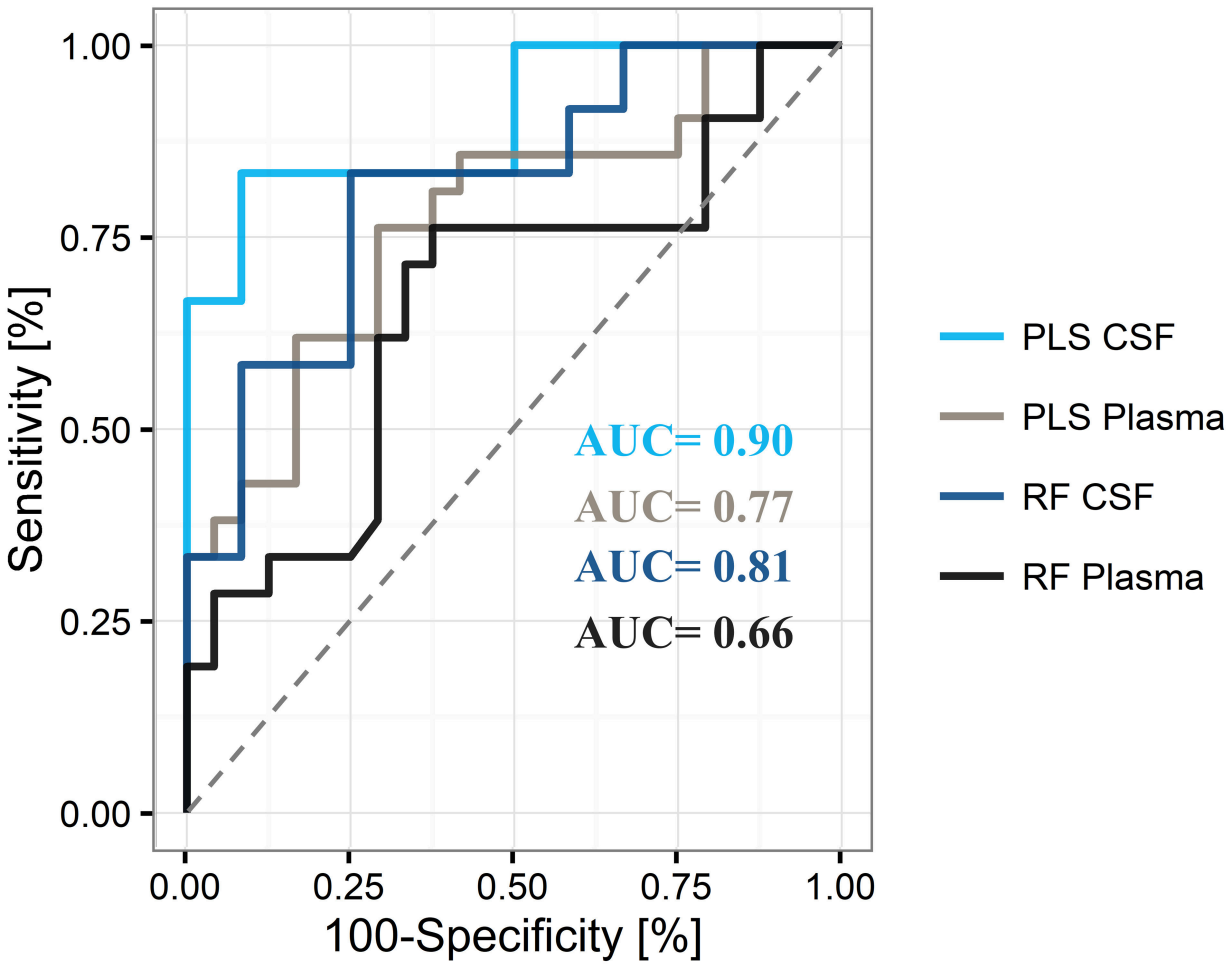
Representative areas under the curve (AUC) for differentiation of Parkinson's disease from controls by use of plasma and cerebrospinal fluid (CSF), and the partial least square (PLS) and random forest (RF) statistical models The PLS model shows superiority over the RF model for the differentiation of states in both compartments.

By using the 20 top-ranked plasma metabolites, we determined an AUC of 0.77 [95% CI = 0.51–0.80, positive predictive value (PPV) = 0.68, negative predictive value (NPV) = 0.65] for the final PLS model and an AUC of 0.66 (95% CI = 0.49–0.78, PPV = 0.63, NPV = 0.65) for the final RF model in the plasma data (Figure 4). The use of 14 CSF metabolites led to an AUC of 0.90 (95% CI = 0.58–0.93, PPV = 0.77, NVP = 0.82) for the tested PLS model and an AUC value of 0.81 (95% CI = 0.49–0.87, PPV = 0.77, NPV = 0.67) for the tested RF model.

Overall, these results indicate superior performance of PLS compared to RF. In addition, the obtained PLS-CSF model showed a higher sensitivity of 0.83 and specificity of 0.75 compared to the PLS-plasma model (sensitivity = 0.62, specificity = 0.71), which indicates a stronger discriminative power of CSF samples compared to plasma samples with a trend toward increased PPV for correct PD classification. A detailed list of model training and test cohort results in plasma and CSF can be found in Supplementary Table 2. Moreover, Monte-Carlo cross validation of the PLS models revealed, that all metabolites were needed to achieve the highest AUC and accuracy in plasma and CSF, respectively (Supplementary Figure 8).

### Pathway analysis reveals multiple altered pathways in PD patients

**Figure 5:**
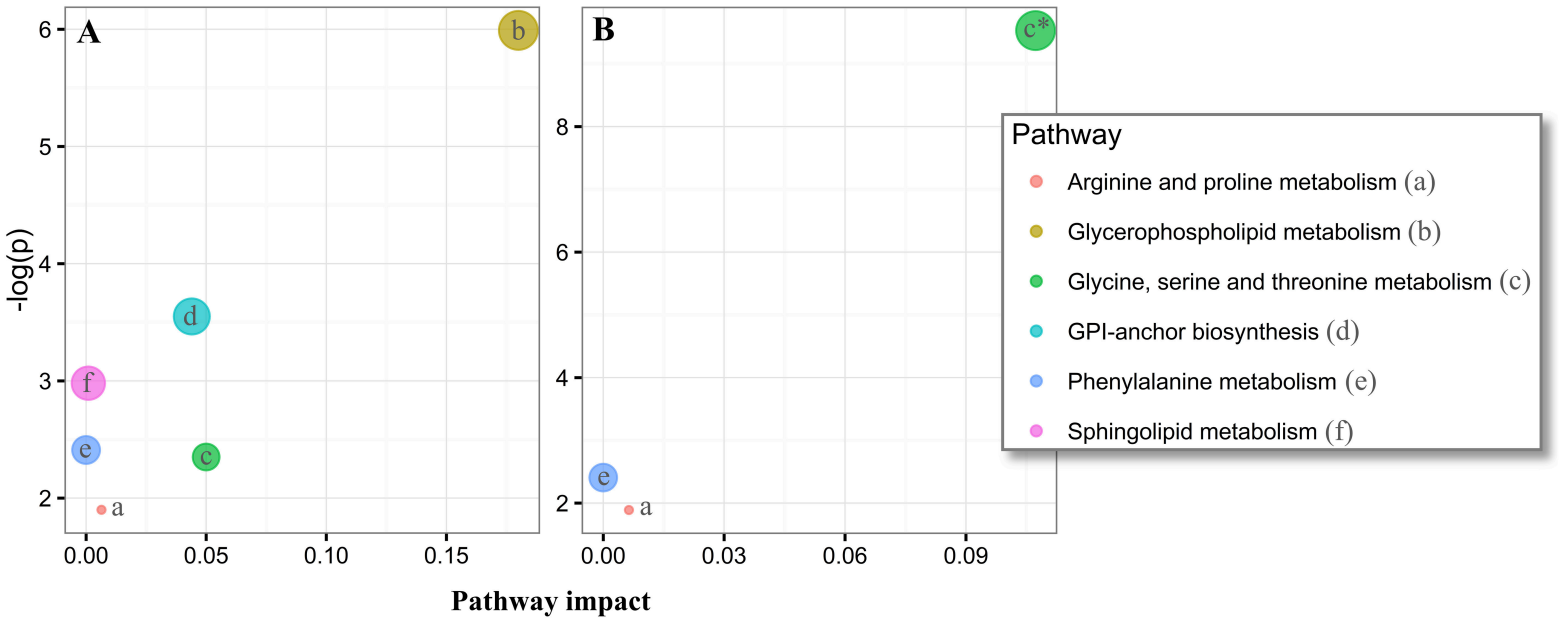
Pathway analysis of altered metabolites in the plasma and cerebrospinal fluid (CSF) of Parkinson's disease (PD) using MetaboAnalyst. The significantly dysregulated metabolites in PD, identified in plasma samples (N metabolites = 20, **A**) and CSF (N metabolites = 14, **B**) were subjected to MetaboAnalyst (http://www.metaboanalyst.ca/) (Xia et al., 2015), to assess associations of respective metabolites to defined pathways. ^*^P < 0.05 after FDR correction. Circle extent (larger) correlates to *p*-value (lower).

Overall, our untargeted metabolic profiling revealed several perturbations, which allowed identification of multiple altered biochemical pathways. The most obvious alterations in plasma were identified in the glycerophospholipid metabolism (*p* = 0.002, FDR-corrected *p* = 0.20, Figure 5A). In CSF, we could identify a perturbed glycine, serine and threonine metabolism (*p* = 7.4 × 10^5^, FDR-corrected *p* = 0.006, Figure 5B).

## Discussion

PD, one of the most common neurodegenerative diseases, shows high clinical variability, making clinical diagnosis often challenging, particularly at the early disease stages when neuromodulatory treatment may be most effective (Davie, 2008). Currently, no reliable molecular biomarker-based diagnosis is available and molecular mechanisms of the disease are still poorly understood. Therefore, molecular biomarkers (or biomarker panels) for PD diagnosis and a better understanding of disease pathogenesis are urgently needed.

Metabolomics, in principle, allows for the measuring and quantification of the entire complement of metabolites in biological fluids, which, therefore, is a suitable technology to capture the functional state of the organism at a given time point. Actually, the breadth of metabolite detection is limited by the ability to annotate measurement spectra, which often results in sets typically comprising a few 100 metabolites. Nonetheless, in combination with machine learning algorithms, such as PLS and RF, differential levels of metabolites between healthy and diseased states can be identified. These metabolites may serve as biomarkers (Gerlach et al., 2012) and lead to a better understanding of novel pathways involved in disease pathogenesis.

Our untargeted metabolomics approach led to the identification of 334 blood plasma and 302 CSF metabolites in controls and early PD patients. The generated representative PLS models in the plasma and CSF cohort showed a high discriminative power between PD patients and controls. Overall, the PLS models outperformed the RF models in both plasma and CSF with higher overall AUC measures. A subset of 20 plasma metabolites and 14 CSF metabolites were defined in a training set of PD patients and controls, and retrieved models differentiated the remaining and independent test set of PD patients and controls with an AUC of 0.77 in plasma and 0.90 in CSF. Interestingly, most of these metabolites were also contained amongst the highest ranked metabolites in the RF models, which underlines the robustness of our findings. Although CSF is less accessible than plasma it is seemingly a more reliable source for promising PD biomarkers, most likely due to its closer proximity to the brain and to the local neurodegenerative process (Botas et al., 2015).

### Glycerophospholipid and sphingolipid metabolism

Our metabolite-based biomarker analysis in combination with PLS machine learning and RF algorithms for PD diagnosis informs on aspects of the pathophysiology of the disease by the identification of alterations in distinct metabolite pathways. In plasma samples, we identified alterations in several lipid classes, including two increased phosphatidylcholines (PC) annotated as PC(44:6) and PC(44:5) and decreased levels of PC(35:6) in PD, phosphatidylethanolamines as indicated by depleted levels of PE(34:1) in PD patients, platelet activating factors (PAF) as indicated by elevated levels of Lyso-PAF-C-16 in PD patients and several sphingolipids, e.g., sphingomyelins (SM), as found by decreased levels of SM(d39:1), SM(d30:1) and SM(d32:1) in PD.

PC lipids and a wide range of phospholipids such as PEs and sphingolipids compromise the majority of eukaryotic cellular neuronal membranes such as the myelin sheath (DeVries et al., 1981; Farooqui et al., 2000; van Meer et al., 2008; Witte et al., 2010). Enzymatic modifications of glycerophospholipids and sphingolipids by phospholipases and nonenzymatic oxidative stress produces a wide range of secondary messengers including PAF and PAF-like lipids (Farooqui et al., 2000, 2007). PAF is known for its contribution to inflammatory responses in the brain (Bazan, 2006) and elevated levels are associated with the central nervous system pathophysiology (McGeer and McGeer, 1995). In addition, sphingomyelins are highly enriched in neuronal cells, exert important biological functions, and are essential for the functionality of the nervous system (Piccinini et al., 2010). Relative changes of glycerophospholipid subclasses have been shown to be associated with neuronal death in PD (Farooqui et al., 2006). Perturbations in sphingolipid metabolism have been found responsible for misfolding events causing the formation of disease-specific protein isoforms such as alpha-synuclein in PD (Jazvinscak Jembrek et al., 2015), amyloid-beta in AD (Mielke et al., 2014) and huntingtin in Huntington's disease (Piccinini et al., 2010). Altered structures of sphingolipids have been described to directly interact with alpha-synuclein in PD (Piccinini et al., 2010) and AD (Mielke et al., 2014). Catabolism of glycerophospholipids generates ceramides and other metabolites that modulate phospholipase activity, which, in turn, produces lipids that can modulate e.g., sphingomyelinase activity (Farooqui et al., 2007). Our findings of elevated levels of Lyso-PAF C-16, PC(44:6), PC(44:5) and depleted levels of PE(34:1), PC(35:6), ethanolamine, which also appears to be depleted in AD (Ellison et al., 1987) and three sphingomyelins in the plasma of our PD cohort support previous results about a perturbed glycerophospholipid and sphingolipid metabolism in PD (Ahmed et al., 2009; Kori et al., 2016) and underlines the potential of these parameters to serve as components of a biomarker panel in PD and to add to a better understanding of disease pathogenesis. Of note, since plasma phospholipids have also been identified in antecedent memory impairment in older adults (Mapstone et al., 2014) their specificity toward PD should be addressed in future studies. Even though, PD has been found to be closely linked to Gaucher's disease (GD), a disease of the lipid metabolism, being involved in Lewy body pathology, the latter pathology is the hallmark of PD. The underlying mutation of the GBA gene causes enhanced phospholipid metabolism and therefore, mutation of the GBA gene could lead to the increased levels of glycerophospholipids in plasma (Brockmann and Berg, 2014). However, all PD patients analyzed in this study were tested negative for this mutation.

In line with the above-mentioned results, we could detect increased levels of 3-ketosphingosine in PD CSF. 3-Ketosphingosine is part of the ceramide metabolism and is formed by the conjunction of serine and palmitoyl-CoA by serine-palmitoyltransferase; the latter is a key enzyme of sphingolipid- and ceramide metabolism and has been reported to be up-regulated in AD (Cutler and Mattson, 2001; Hanada, 2003; Wood, 2012) and in association with altered ceramide metabolism in PD such as caused by GBA mutations (Mielke et al., 2013) and in other Lewy body diseases (Bras et al., 2008).

### Fatty acid oxidation

Moreover, we detected elevated levels of the acylcarnitine annotated as o-adipoylcarnitine in CSF. Brain acylcarnitines support lipid biosynthesis and activity of antioxidants; they also enhance cholinergic neurotransmission (Jones et al., 2010). Increased levels of o-adipoylcarnitine in CSF could be associated with increased activity against oxidative stress perturbed lipid biosynthesis in PD, which needs further investigation. In addition, reduced levels of N-laroylglycine were found in plasma in our PD cohort. This metabolite of the class acylglycine is a minor metabolite of fatty acids and is produced through the action of glycine N-acyltransferase. This fits well with the assumption of relevant perturbations in fatty acid oxidation processes that are associated with this disease (Wilcox et al., 1999; Suhre et al., 2010; Dias et al., 2013; Hwang, 2013; Saiki et al., 2017) and in other neurodegenerative diseases such as amyotrophic lateral sclerosis (ALS) (Pollari et al., 2014) and AD (Selley et al., 2002).

### Glycine, serine, and, threonine metabolism and branched chain fatty acids

Of note, depleted levels of sarcosine, betaine, and dimethylglycine (DMG) in CSF indicate dysregulated glycine, serine, and threonine metabolism in PD, which has previously been reported in PD and ALS (Sertbaş et al., 2014). Interestingly, increased levels of glycine were also found in animal models of PD, such as 6-OHDA-treated mice (Solis et al., 2016). DMG is produced by metabolizing choline into glycine, and is a by-product of homocysteine metabolism where betaine is converted to methionine and DMG by betaine-homocysteine methyltransferase. Studies have shown that DMG decreases oxidative stress (Takahashi et al., 2016), improves immune response (Graber et al., 1981) and acts as anticonvulsant (Freed, 1985). Of note, the major portion of glycine and serine synthesis occurs in hepatic tissue via the “phosphorylated pathway,” therefore corresponding changes in brain may not be detectable in plasma. In addition, depleted levels of S-(2-methylpropionyl)-dihydrolipoamide-E, as observed in our PD CSF samples, have not yet been reported by other groups. This observation suggests perturbations in valine, leucine, and isoleucine degradation pathways to be associated with PD as this metabolite is found in the second to last step in the synthesis of these branched chain fatty acids via the enzyme 2-oxoisovalerate dehydrogenase. It is then converted to isobutyryl-CoA via the enzyme dihydrolipoyllysine-residue (2-methylpropanoyl)transferase. However, none of these enzymes have yet been reported to be associated with PD or other neurodegenerative diseases. An association was found with progressive neurodegeneration in maple syrup urine disease (Chuang et al., 1994).

### Phenylalanine and arginine proline metabolism and glycine biosynthesis and degradation

We identified increased levels of sarcosine, the N-methyl derivative of glycine, in the plasma of our PD cohort. Our result is consistent with previous findings in plasma (Antonio Molina et al., 2017). Sarcosine is associated to the phenylalanine and arginine proline metabolisms, and it is involved in glycine biosynthesis and degradation. Interestingly, this metabolite was decreased in our PD CSF samples. To the best of our knowledge, decreased levels of sarcosine in the CSF of PD patients have not been described previously and underlying mechanisms in the neurodegenerative process remain unclear.

### Gut microbiota and neurodegeneration

The potential marker annotated as alpha-N-phenylacetyl-L-glutamine (phenylacetylglutamine) was highly elevated in the plasma and CSF of PD patients. This metabolite most probably originates from the putrefaction of phenylalanine and tyrosine by the gut microbiota (Swann et al., 2013). Interestingly, the involvement of the gut microbiota has been suggested as one of the key factors of neurodegeneration in PD (Houser and Tansey, 2017; Marizzoni et al., 2017).

### Other metabolites

Our untargeted metabolomics approach revealed some additional, statistically significant changes in metabolite concentrations between PD patients and controls in both plasma and CSF, which need further investigation. First, the urinary metabolite annotated as 1,3-dimethyluracil was increased in PD plasma. 1,3-dimethyluracil is a methyl derivative of urate, and previous studies have reported lowered levels of urate in PD serum (Andreadou et al., 2009). Although high urate levels in the blood have been associated with lower risks of developing PD (Cipriani et al., 2010) and urate may protect from fast clinical progression in PD (Ascherio et al., 2009), AD and ALS (Paganoni and Schwarzschild, 2017), the specific role of 1,3-dimethyluracil in PD remains to be investigated. Second, we identified several significantly altered levels of di-peptides as, e.g., arg-ala (increased in PD plasma), ser-glu and prolyl-tyrosine (increased in PD CSF). Altered levels were also observed for the tri-peptide depleted leu-lrp-trp (depleted in PD CSF). At this point of time, it is unclear whether these changes are caused by altered protein degradation or altered amino acid metabolism, and if these peptides have neurotransmitter functions, which could then possibly explain clinical features of the disease. Finally, we observed increased CSF levels of (+)-gamma-hydroxy-l-homoarginine, which have again not yet been reported in PD or any other neurodegenerative disease and need further investigation.

The study presented here informs about previously unreported marker candidates [e.g., various sphingo- and glycerophospholipids, N-laroylglycine, 1,3-dimethyluracil, phenylacetylglutamine, 3-ketosphingosine, O-adipoylcarnitine, S-(2-methylpropionyl)-dihydrolipoamide-E, (+)-gamma-hydroxy-l-homoarginine and several short chain peptides], and did not confirm all significant results reported in the previous studies (e.g., increased levels of fructose, mannose und threonic acid and decreased levels of dehydroascorbic acid in early stage PD patients Trezzi et al., 2017).

The main aim of this study is to provide additional original data from a highly specific and well-defined cohort of early PD, using also a high-quality approach of metabolomics analyses, but not to provide an exhaustive and systematic meta-analysis of all metabolomics data currently available for PD diagnosis. Still, heterogeneity across previous studies and this study may be due to, e.g., differencing cohorts including distinct recruitment strategies, different analytical platforms (which to most extent rely on reversed phase chromatography), and differences in targeted assays and GC/MS profiling (Supplementary Table 3). In addition, different protocols for metabolite extraction, differing biological matrices, data exploration and statistical analysis will bias the results [for similar problems in AD see (Gonzalez-Dominguez et al., 2017)] and should be considered when comparing such results.

As the current study aimed at differentiating PD from age-matched controls, further studies are needed to investigate whether observed changes are PD-specific. Replication in an independent larger cohort, and differentiation to other disorders, specifically progressive supranuclear palsy (PSP), corticobasal degeneration (CBD), and multiple system atrophy (MSA) in the very early stages of the diseases are needed.

## Conclusion

In conclusion, (i) metabolic profiling of plasma and CSF samples in combination with machine learning analysis was found to be a promising approach for a limitedly-invasive diagnosis of PD, (ii) our pilot study corroborates previous studies seeing altered glycerophospholipid, sphingolipid, and amino acid metabolisms as relevant mechanisms of PD pathogenesis, and (iii) specifically the identification of novel and partly unknown metabolites require further investigation in independent cohorts using also longitudinal approaches.

## Data availability statement

### Restrictions apply to the datasets

The datasets for this manuscript are not publicly available because of the specific structure of the study: the ethical proposal of the Neuro Biobank Tübingen does not include such an option. Requests to access the datasets should be directed to Daniel Stoessel.

## Author contributions

DaS, CS, NS, AN, DB, and WM: designed the study; DaS: conducted the experiments; DaS, CS, MT, DaS, IR-M, CD, DW, DB, and WM: acquired and analyzed data; DiS, NS, DB, AN, and WM: supervised the project; DaS and WM: drafted, and all authors revised the manuscript.

### Conflict of interest statement

The authors declare that the research was conducted in the absence of any commercial or financial relationships that could be construed as a potential conflict of interest.

## Supplementary material

Supplementary Figure 1 Score distance and orthogonal distance matrix used for outlier detection. Samples in the highlighted area were considered outliers in **(A)** plasma and **(B)** CSF. C, controls; PD, Parkinson's disease.

Supplementary Figure 2 Intensity levels for potential PD plasma marker panel determined by our PLS model showing relative differences in abundance in each patient analyzed. Red, controls; gray, Parkinson's disease (PD) patient. ^*^Statistically significant change according to Welch's *t*-test statistics or Wilcoxon test (*p*-value < 0.05) after FDR correction.

Supplementary Figure 3 Top 100 proposed metabolites in the plasma PLS model. Each individual scaled VIP score plotted from the highest to the lowest value. Dashed line: cut-off used to determine most influential metabolites in the model based on the point where the slope flattens (threshold = 68).

Supplementary Figure 4 Top 100 proposed metabolites in the plasma RF model. Each individual scaled importance score plotted from the highest to lowest value. Dashed line: cut-off used to determine most influential metabolites in the model based on the point where the slope flattens (threshold = 59).

Supplementary Figure 5 Intensity levels for potential PD CSF marker panel determined by our PLS model showing relative differences in abundance in each patient analyzed. Red: controls, light blue: Parkinson's disease (PD) patient. ^*^Statistically significant change according to Welch's t-test statistics or Wilcoxon test (*p*-value < 0.05) after FDR correction. ^**^Statistically significant change according to Welch's t-test statistics or Wilcoxon test (*p*-value < 0.01) after FDR correction.

Supplementary Figure 6 Top 100 proposed metabolites in the CSF PLS model. Each individual scaled VIP score plotted from the highest to lowest value. Dashed line: cut-off used to determine most influential metabolites in the model based on the point where the slope flattens (threshold = 69).

Supplementary Figure 7 Top 100 proposed metabolites in the CSF RF model. Each individual scaled importance score plotted from the highest to lowest value. Dashed line: cut off used to determine most influential metabolites in the model based on the point where the slope flattens (threshold = 55).

Supplementary Figure 8 Two component PLS Monte Carlo cross validation models for plasma and CSF. Corresponding ROC curves of different metabolite sets (2, 3, 5, 7, 10, 20) in **(A)** plasma and (2, 3, 5, 7, 10, 14) **(B)** CSF. Predicted accuracy with different number of metabolites in **(C)** plasma and **(D)** CSF. Abbreviations: Var., variable e.g., metabolite, AUC, area under the curve; CI, 95% confidence interval.

Supplementary Table 1 PD_Metabolome. List of identified putative metabolites from untargeted profiling in PD plasma (Sheet plasma) and PD CSF (Sheet CSF) (^*^.xlsx file).

Supplementary Table 2 Model parameters obtained for the PLS and RF models in plasma and CSF.

Supplementary Table 3 Current state of metabolomics research in Parkinson's disease.
