# Supplementary material for: Promising Metabolite Profiles in the Plasma and CSF of Early Clinical Parkinson's Disease
Source: Front Aging Neurosci. 2018 Mar 5;10:51. doi: 10.3389/fnagi.2018.00051 (PMC5844983; doi:10.3389/fnagi.2018.00051)
Supplement: Supplementary Figure 1 — Score distance and orthogonal distance matrix used for outlier detection. Samples in the highlighted area were considered outliers in (A) plasma and (B) CSF. C, controls; PD, Parkinson's disease. [file DataSheet1.pdf]

Supplemental material to:

Metabolite profiles in the plasma and CSF of early clinical Parkinson's disease

Daniel Stoessel, Claudia Schulte*, Marcia Cristina Teixeira dos Santos, Dieter Scheller, Irene Rebollo-Mesa, Christian Deuschle, Dirk Walther, Nicolas Schauer, Daniela Berg, Andre Nogueira da Costa, Walter Maetzler

*** Correspondence:** claudia.schulte@uni-tuebingen.de

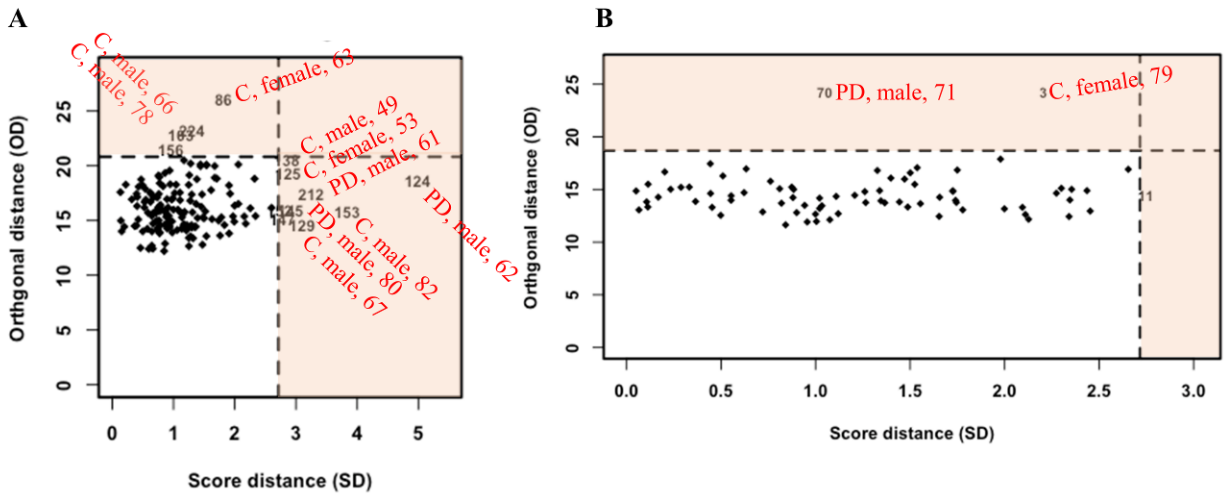

Supplementary Figure 1: Score distance and orthogonal distance matrix used for outlier detection. Samples in the highlighted area were considered outliers in (**A**) plasma and (**B**) CSF. C = controls, PD = Parkinson's disease

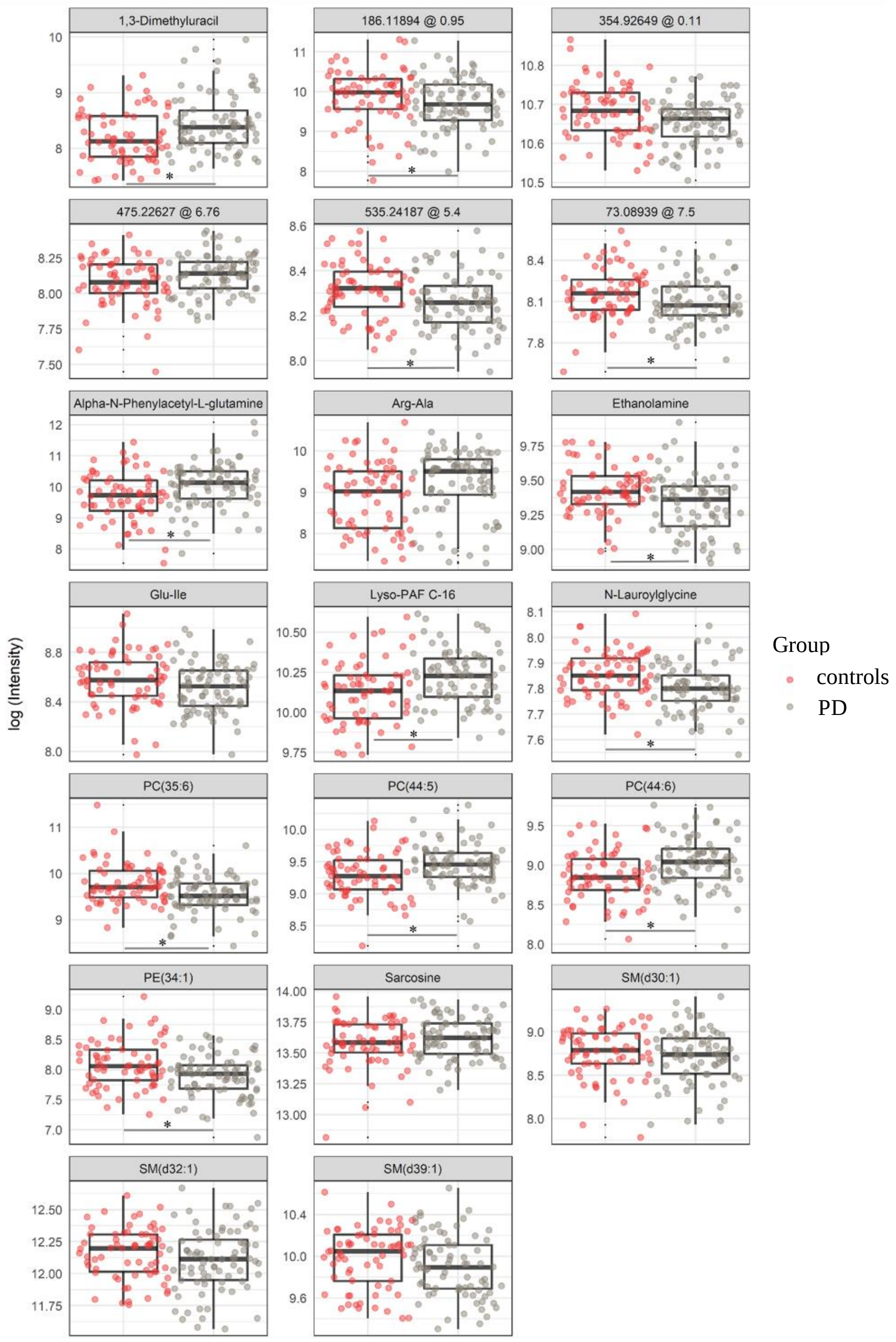

Supplementary Figure 2: Intensity levels for potential PD plasma marker panel determined by our PLS model showing relative differences in abundance in each patient analysed. Red: controls, grey: Parkinson's disease (PD) patient. * Statistically significant change according to Welch's t-test statistics or Wilcoxon test (p-value < 0.05) after FDR correction.

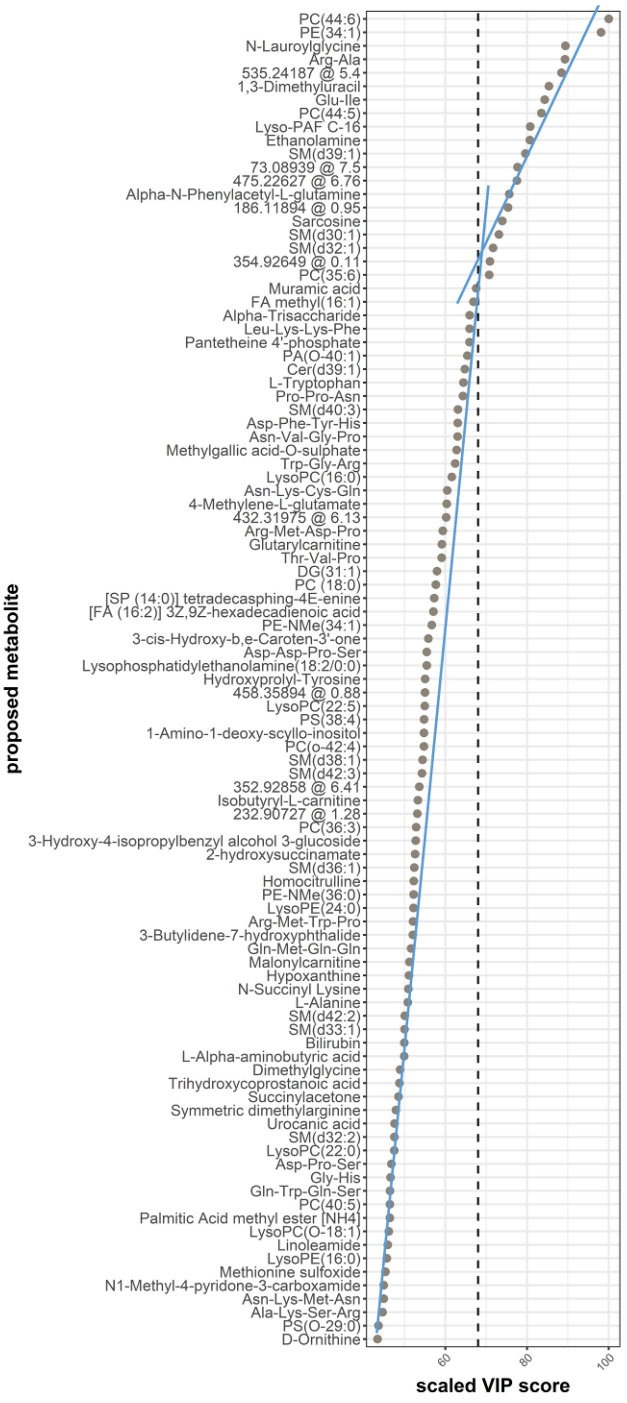

Supplementary Figure 3: Top 100 proposed metabolites in the plasma PLS model. Each individual scaled VIP score plotted from the highest to the lowest value. Dashed line: cut-off used to determine most influential metabolites in the model based on the point where the slope flattens (threshold = 68).

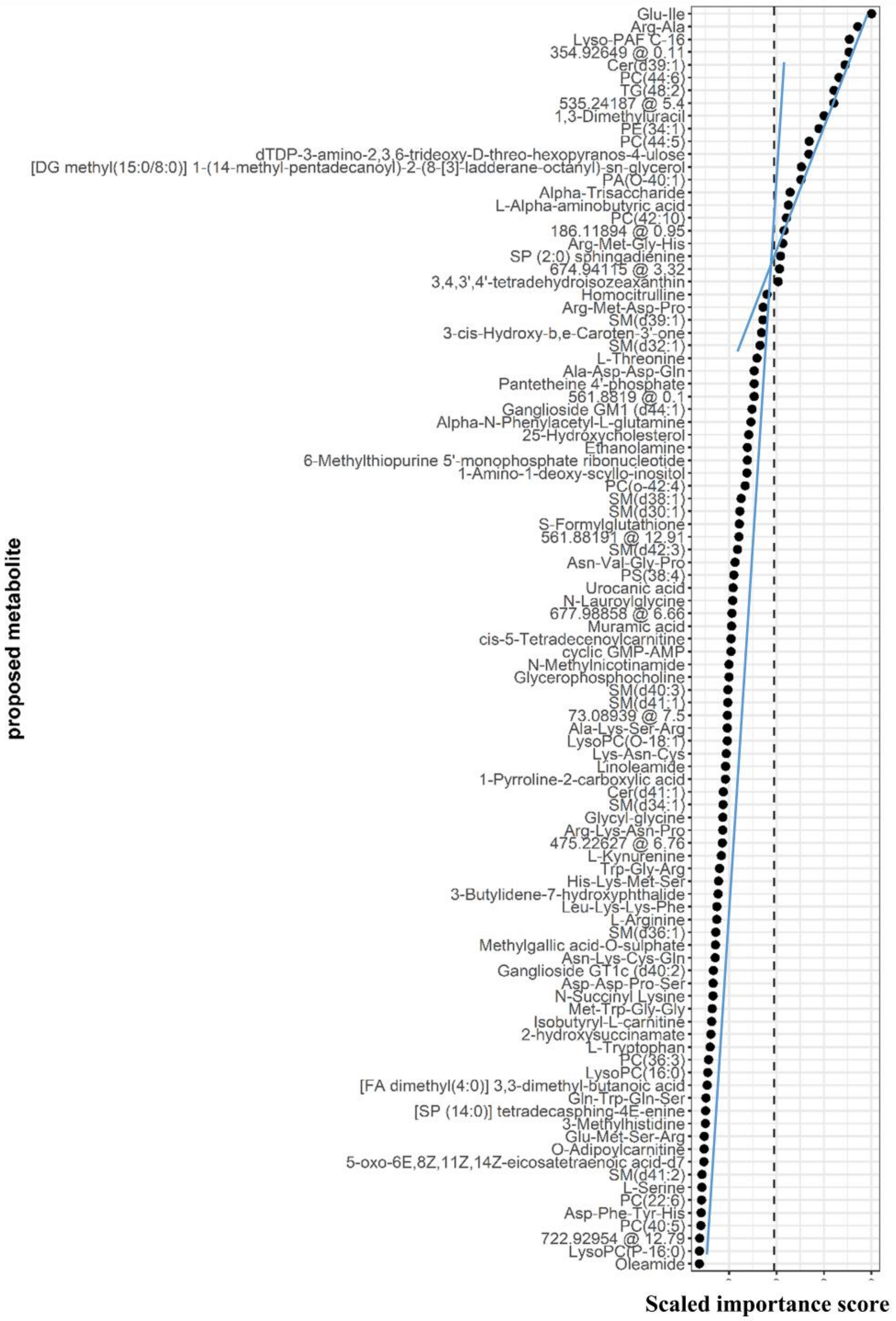

Supplementary Figure 4: Top 100 proposed metabolites in the plasma RF model. Each individual scaled importance score plotted from the highest to lowest value. Dashed line: cut-off used to determine most influential metabolites in the model based on the point where the slope flattens (threshold = 59).

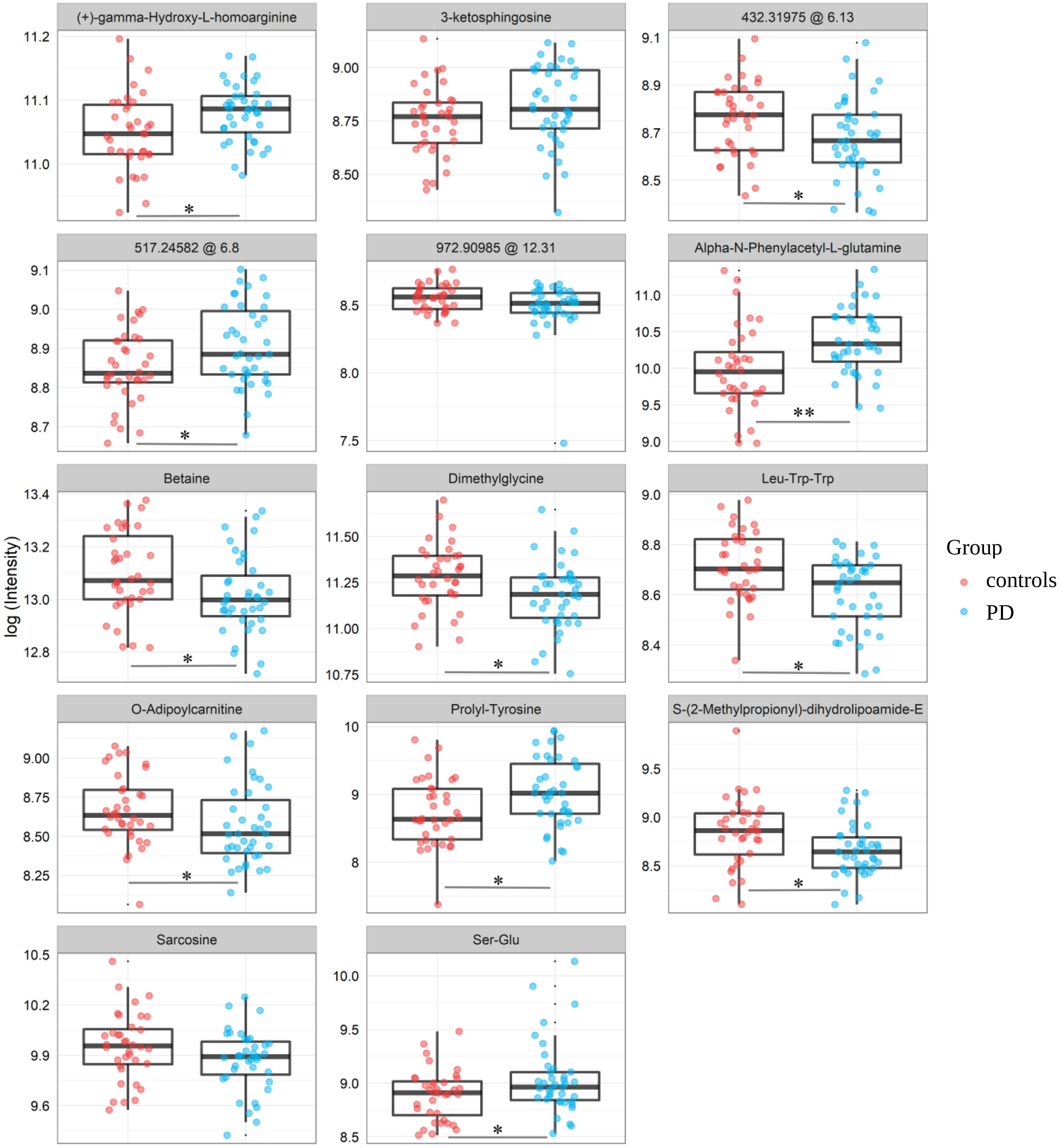

Supplementary Figure 5: Intensity levels for potential PD CSF marker panel determined by our PLS model showing relative differences in abundance in each patient analysed. Red: controls, light blue: Parkinson's disease (PD) patient. * Statistically significant change according to Welch's t-test statistics or Wilcoxon test (p-value < 0.05) after FDR correction. ** Statistically significant change according to Welch's t-test statistics or Wilcoxon test (p-value < 0.01) after FDR correction.

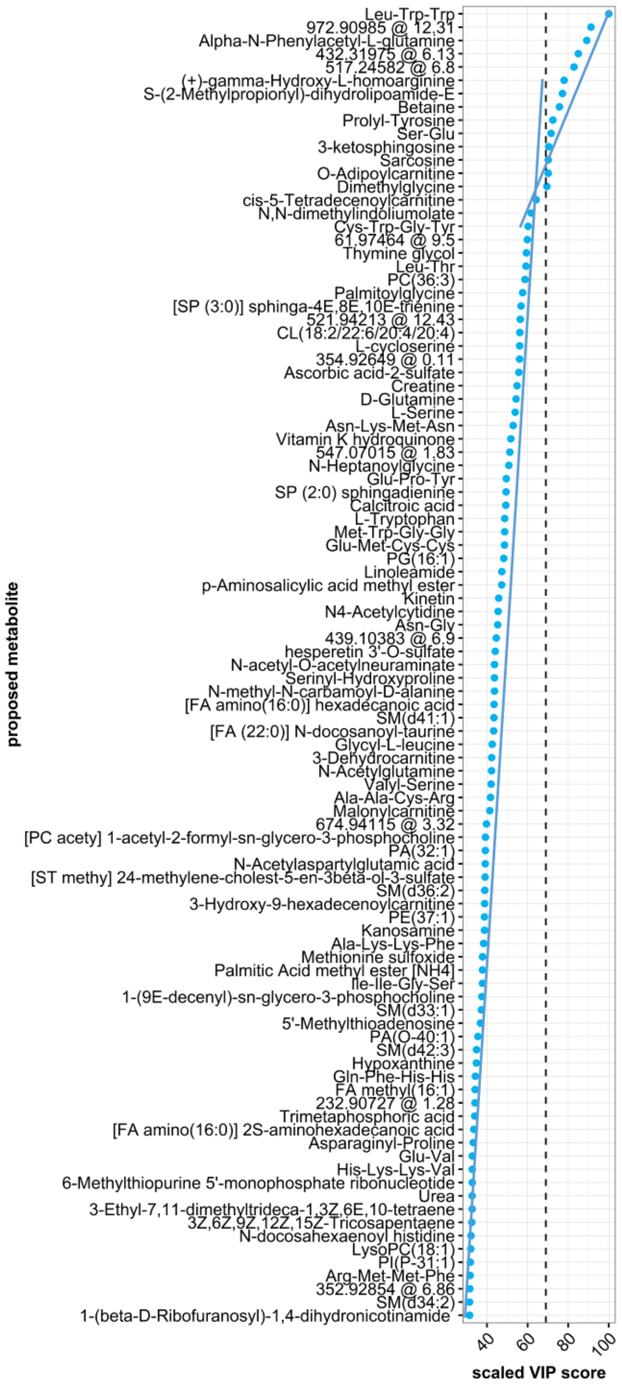

Supplementary Figure 6: Top 100 proposed metabolites in the CSF PLS model. Each individual scaled VIP score plotted from the highest to lowest value. Dashed line: cut-off used to determine most influential metabolites in the model based on the point where the slope flattens (threshold = 69).

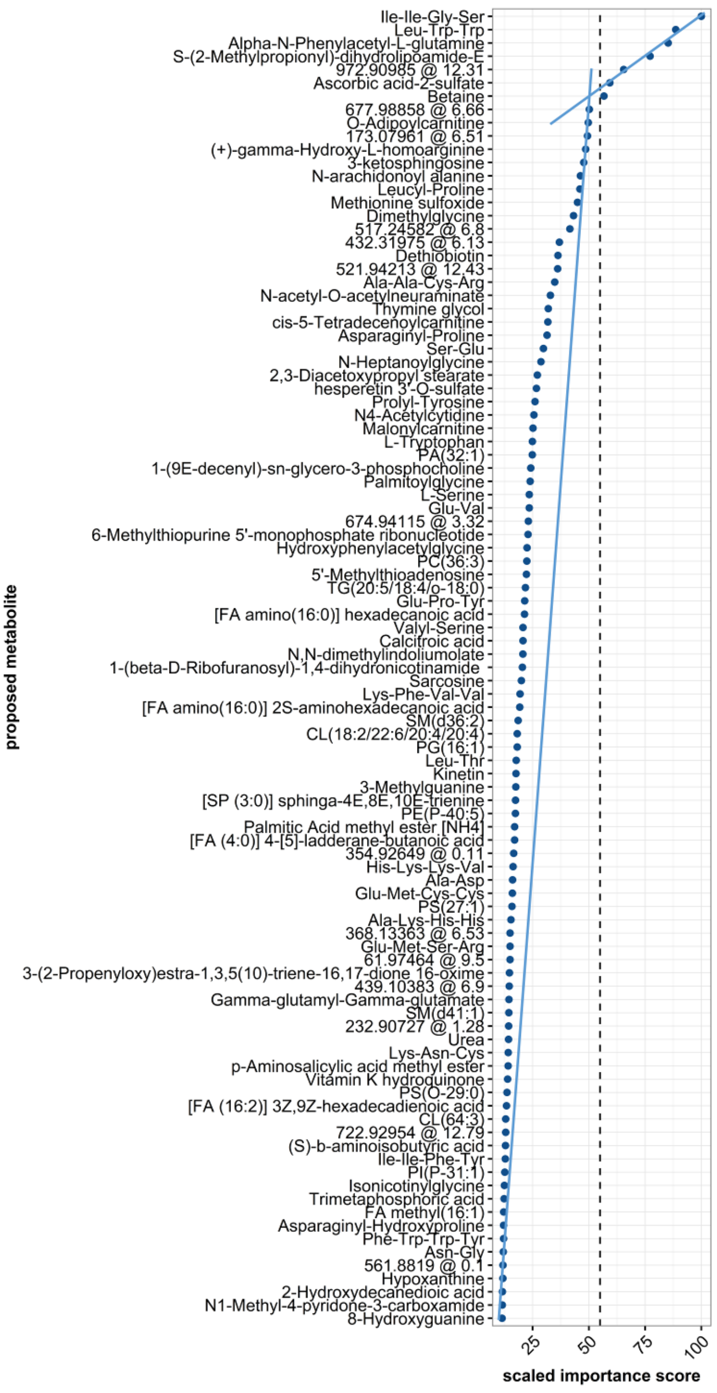

Supplementary Figure 7: Top 100 proposed metabolites in the CSF RF model. Each individual scaled importance score plotted from the highest to lowest value. Dashed line: cut off used to determine most influential metabolites in the model based on the point where the slope flattens (threshold = 55).

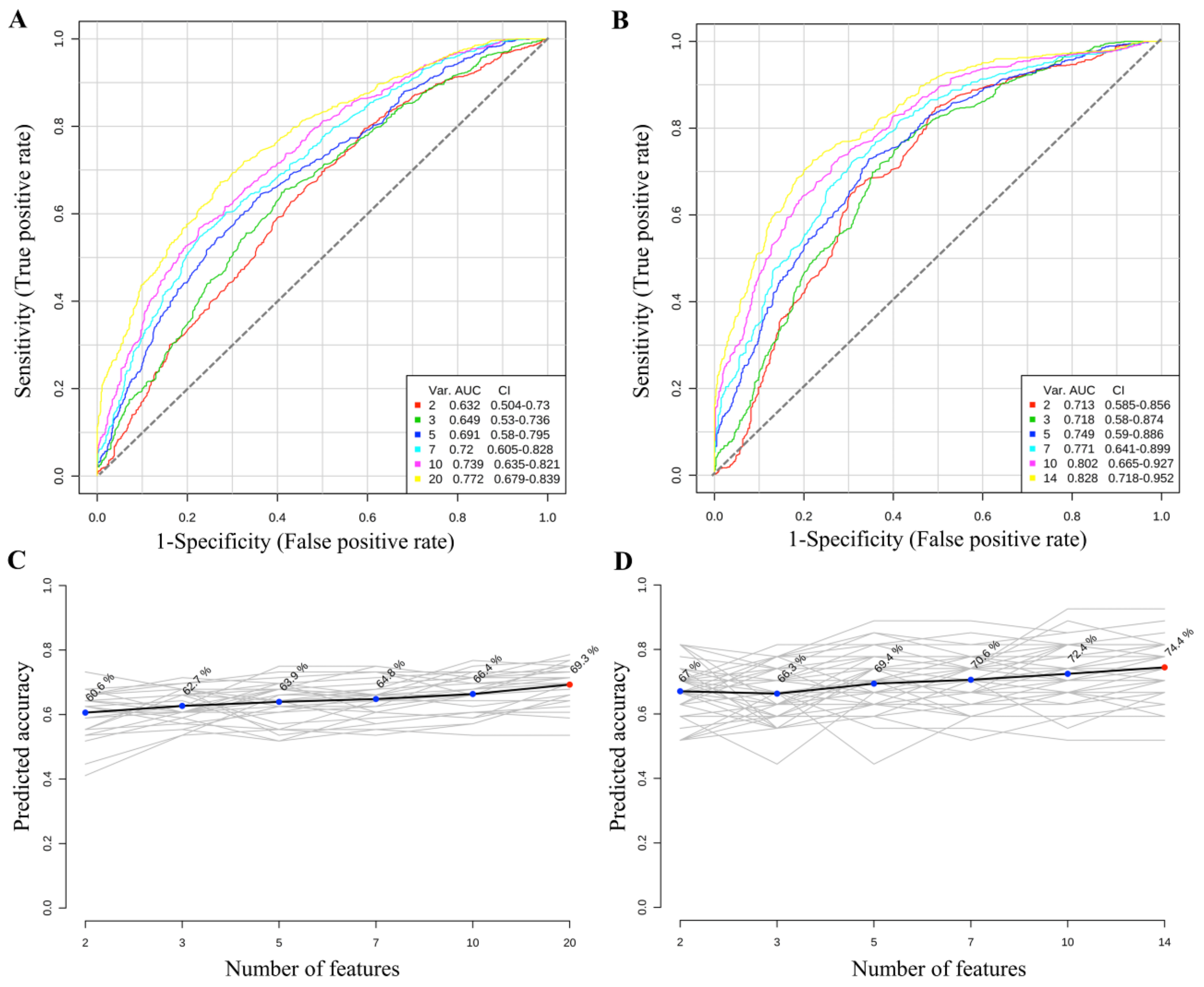

Supplementary Figure 8: Two component PLS Monte Carlo cross validation models for plasma and CSF. Corresponding ROC curves of different metabolite sets (2, 3, 5, 7, 10, 20) in (**A**) plasma and (2, 3, 5, 7, 10, 14) (**B**) CSF. Predicted accuracy with different number of metabolites in (**C**) plasma and (**D**) CSF. Abbreviations: Var. = variable e.g. metabolite, AUC = area under the curve, CI = 95% confidence interval.

Supplementary Table 2: Model parameters obtained for the PLS and RF models in plasma and CSF.

Parameter	PLS Plasma	RF Plasma	PLS CSF	RF CSF
Sensitivity train	0.68	0.67	0.77	0.81
Specificity train	0.80	0.76	0.83	0.87
AUC train	0.82	0.81	0.88	0.91
Sensitivity test	0.62	0.57	0.83	0.58
Specificity test	0.71	0.71	0.75	0.83
PPV	0.68	0.63	0.77	0.77
NPV	0.65	0.65	0.82	0.67
Accuracy test	0.67	0.64	0.79	0.71
AUC test	0.77	0.66	0.90	0.81
P-Value [Acc > NIR]	0.049*	0.087	0.003*	0.032*
95% CI	0.51-0.8	0.49-0.78	0.58-0.93	0.49-0.87

PPV = Positive Predictive Value, NPV = Negative Predictive Value. 95% CI of to the overall accuracy rate of the model calculated by using a binomal test. * p-value <0.05. Acc = accuracy, NIR = no information rate (largest class percentage in the data e.g. the proportion of classes correctly classified by chance).

Supplementary Table 3: Current state of metabolomics research in Parkinson's disease

Study	Matrix	Cohort	Platform	Findings in PD
Han et al., 2017	Serum	PD (*n* = 43)<br>PDD (*n* = 16)<br>controls (*n* = 42)	CIL-LC/MS	3-hydroxykynurenine and methionine sulfoxide ↑; vanillylmandelic acid ↑; theophylline, 5-acetylamino-6-amino-3-methyluracil, xanthine and citrulline↓
Saiki et al., 2017	Serum	PD (*n* = 254)<br>controls (*n* = 77)	LC/MS<br>CE/MS	3-Methoxytyrosine, urea, homovanillic acid, guanidinosuccinic acid, cortisone, oleoylethanolamine, palmitoylethanolamine, citric acid and deoxycholic acid ↑; long-chain acylcarnitines ↓
LeWitt et al., 2017	Plasma<br>CSF	PD (*n* = 49) (d) *	GC/MS	medium-long chain fatty acids, aspartylphenylalanine, benzoate, serine ↑ (Plasma); inosine ↓ (Plasma)
Havelund et al., 2017	Plasma<br>CSF	PD (*n* = 26)<br>PDd (*n* = 10)<br>controls (*n* = 14)	LC/MS	3-hydroxykynuerine, kynurenic acid ↑ (Plasma); anthranilic acid ↓ **(**Plasma & CSF**)**
Burte et al., 2017	Plasma	PD (*n* = 41)<br>controls (*n* = 40)	LC/MS	Acylcarnitine ↑, 1-methylhistamine ↓
Trezzi et al., 2017	CSF	ePD (*n* = 44) (d)<br>controls (*n* = 43)	GC/MS	Fructose, mannose, and threonic acid ↑; dehydroascorbic acid ↓
Wuolikainen et al., 2016	Plasma<br>CSF	PD (*n* = 22)<br>controls (*n* = 28)<br>ALS (*n* = 22)	LC/MS<br>GC/MS	Alanine, leucine, isoleucine ↓ (Plasma & CSF)
Hatano et al., 2016	Serum	PD (*n* = 35)<br>controls (*n* = 15)	LC/MS<br>GC/MS	Tryptophan, caffeine and its metabolites, bilirubin and ergothioneine ↓; levodopa metabolites and biliverdin ↑
Luan et al., 2015<br>Luan et al., 2015a	Urine	PD (*n* = 92)<br>PD (*n* = 108)<br>controls (*n* = 65)<br>controls (*n* = 104)	LC/MS<br>GC/MS	Pathway variations in branched chain amino acid metabolism, glycine derivation, steroid hormone biosynthesis, tryptophan metabolism, and phenylalanine metabolism
Ohman and Forsgren, 2015	CSF	PD (*n* = 10)<br>controls (*n* = 10)	H-NMR	Alanine, creatinine and mannose ↓
Trupp et al., 2014	Plasma<br>CSF	PD (*n* = 20)<br>controls (*n* = 20)	GC/MS	Methionine, threonine, alanine, serine, pyroglutamate and ketoleucine ↑ (Plasma); creatinine and tryptophane ↓ (CSF); C16 and C18 fatty acids ↓ (Plasma)
Lewitt et al., 2013	CSF	PD (*n* = 48)<br>controls (*n* = 57)	LC/MS<br>GC/MS	3-hydroxykynuerine and kynurenic acid ↑; acetylated amino acids and GSSG ↓
Roede et al., 2013	Serum	rPD (*n* = 39)<br>sPD (*n* = 41)<br>controls (*n* = 20)	LC/MS	N8-acetylspermidine ↑
Ahmed et al., 2009	Plasma	PD (*n* = 43) (d)<br>controls (*n* = 37)	H-NMR	Pyruvate, sorbitol, myoinositol, ethymalonate and propylene glycol ↑; suberate, methylmalonate, galactitol, citrate, malate, succinate, glycerol, isocitrate, ethanolamine, ascorbate, threonate, gluconate, acetate, trimethylamine, glutarate, methylamine and glucolate ↓
Johansen et al., 2009	Plasma	PD (*n* = 41)<br>LRRK2 (*n* = 12)<br>controls (*n* = 20)	LCECA	Hypoxanthine and other purines ↓
Michell et al., 2008	Serum<br>Urine	PD (*n* = 23) (f)<br>controls (*n* = 23)<br>(f)	GC/MS	Various monosaccharides, sugar alcohol, suberic acid (urine) ↑; D, 2-mercapto-4,6-diaminopyridine, Octenoic acid, Urea ↓
Bogdanov et al., 2008	Plasma	PD (*n* = 60)<br>controls (*n* = 25)	LCECA	8-hydroxy-2-deoxyguanosine ↑; uric acid and glutathione ↓

Abbreviations: ePD = early stage PD, rPD = rapid progress PD, sPD = slow progress PD, PDd = Parkinson's disease patients receiving L-DOPA, CIL-LC/MS = Chemical isotope labeling liquid chromatography mass spectrometry, CE = capillary electrophoresis, GC = gas chromatography, H-NMR = Proton nuclear magnetic resonance, LCECA = high performance liquid chromatography coupled with electrochemical coulometric array detection, CEA = colorimetric enzyme assay, (f) only females, (d) drug naïve. LRKK = PD patients with LRRK2 gene mutation. * collected twice with an interval up to two years.

References

Ahmed, S. S., Santosh, W., Kumar, S., and Christlet, H. T. T. (2009). Metabolic profiling of Parkinson's disease: evidence of biomarker from gene expression analysis and rapid neural network detection. *J. Biomed. Sci.* 16, 63. doi:10.1186/1423-0127-16-63.

Bogdanov, M., Matson, W. R., Wang, L., Matson, T., Saunders-Pullman, R., Bressman, S. S., et al. (2008). Metabolomic profiling to develop blood biomarkers for Parkinson's disease. *Brain* 131, 389–396. doi:10.1093/brain/awm304.

Burte, F., Houghton, D., Lowes, H., Pyle, A., Nesbitt, S., Yarnall, A., et al. (2017). metabolic profiling of Parkinson's disease and mild cognitive impairment. *Mov. Disord.* 32, 927–932. doi:10.1002/mds.26992.

Han, W., Sapkota, S., Camicioli, R., Dixon, R. A., and Li, L. (2017). Profiling novel metabolic biomarkers for Parkinson's disease using in-depth metabolomic analysis. *Mov. Disord.* doi:10.1002/mds.27173.

Hatano, T., Saiki, S., Okuzumi, A., Mohney, R. P., and Hattori, N. (2016). Identification of novel biomarkers for Parkinson's disease by metabolomic technologies. *J. Neurol. Neurosurg. Psychiatry* 87, 295–301. doi:10.1136/jnnp-2014-309676.

Havelund, J. F., Andersen, A. D., Binzer, M., Blaabjerg, M., Heegaard, N. H. H., Stenager, E., et al. (2017). Changes in kynurenine pathway metabolism in Parkinson patients with L-DOPA-induced dyskinesia. *J. Neurochem.* 142, 756–766. doi:10.1111/jnc.14104.

Johansen, K. K., Wang, L., Aasly, J. O., White, L. R., Matson, W. R., Henchcliffe, C., et al. (2009). Metabolomic profiling in LRRK2-related Parkinson's disease. *PLoS One* 4, e7551. doi:10.1371/journal.pone.0007551.

Lewitt, P. A., Li, J., Lu, M., Beach, T. G., Adler, C. H., and Guo, L. (2013). 3-hydroxykynurenine and other Parkinson's disease biomarkers discovered by metabolomic analysis. *Mov. Disord.* 28, 1653–1660. doi:10.1002/mds.25555.

LeWitt, P. A., Li, J., Lu, M., Guo, L., and Auinger, P. (2017). Metabolomic biomarkers as strong correlates of Parkinson disease progression. *Neurology* 88, 862–869. doi:10.1212/WNL.0000000000003663.

Luan, H., Liu, L.-F., Meng, N., Tang, Z., Chua, K.-K., Chen, L.-L., et al. (2015a). LC-MS-based urinary metabolite signatures in idiopathic Parkinson's disease. *J. Proteome Res.* 14, 467–478. doi:10.1021/pr500807t.

Luan, H., Liu, L.-F., Tang, Z., Zhang, M., Chua, K.-K., Song, J.-X., et al. (2015b). Comprehensive urinary metabolomic profiling and identification of potential noninvasive marker for idiopathic Parkinson's disease. *Sci. Rep.* 5, 13888. doi:10.1038/srep13888.

Michell, A. W., Mosedale, D., Grainger, D. J., and Barker, R. A. (2008). Metabolomic analysis of urine and serum in Parkinson's disease. *Metabolomics* 4, 191. doi:10.1007/s11306-008-0111-9.

Ohman, A., and Forsgren, L. (2015). NMR metabonomics of cerebrospinal fluid distinguishes between Parkinson's disease and controls. *Neurosci. Lett.* 594, 36–39. doi:10.1016/j.neulet.2015.03.051.

Roede, J. R., Uppal, K., Park, Y., Lee, K., Tran, V., Walker, D., et al. (2013). Serum Metabolomics of Slow vs. Rapid Motor Progression Parkinson's Disease: a Pilot Study. *PLoS One* 8, e77629. Available at: https://doi.org/10.1371/journal.pone.0077629.

Saiki, S., Hatano, T., Fujimaki, M., Ishikawa, K.-I., Mori, A., Oji, Y., et al. (2017). Decreased long-chain acylcarnitines from insufficient β-oxidation as potential early diagnostic markers for Parkinson's disease. *Sci. Rep.* 7, 7328. doi:10.1038/s41598-017-06767-y.

Trezzi, J.-P., Galozzi, S., Jaeger, C., Barkovits, K., Brockmann, K., Maetzler, W., et al. (2017). Distinct metabolomic signature in cerebrospinal fluid in early parkinson's disease. *Mov. Disord.* 32, 1401–1408. doi:10.1002/mds.27132.

Trupp, M., Jonsson, P., Ohrfelt, A., Zetterberg, H., Obudulu, O., Malm, L., et al. (2014). Metabolite and peptide levels in plasma and CSF differentiating healthy controls from patients with newly diagnosed Parkinson's disease. *J. Parkinsons. Dis.* 4, 549–560. doi:10.3233/JPD-140389.

Wuolikainen, A., Jonsson, P., Ahnlund, M., Antti, H., Marklund, S. L., Moritz, T., et al. (2016). Multi-platform mass spectrometry analysis of the CSF and plasma metabolomes of rigorously matched amyotrophic lateral sclerosis, Parkinson's disease and control subjects. *Mol. Biosyst.* 12, 1287–1298. doi:10.1039/c5mb00711a.
